# China Land Carbon Budget (CLCB1.0): a comprehensive estimate of the land carbon budget in China

**DOI:** 10.1093/nsr/nwaf052

**Published:** 2025-02-19

**Authors:** Jiangzhou Xia, Xiaosheng Xia, Xuhui Wang, Weimin Ju, Zhengyang Lin, Zhangcai Qin, Yuxing Sang, Yanzi Yan, Wenping Yuan, Xu Yue, Haicheng Zhang, Hao Zhou, Qiuan Zhu

**Affiliations:** Tianjin Key Laboratory of Water Resources and Environment, Tianjin Normal University, China; School of Atmospheric Sciences, Sun Yat-sen University, China; Institute of Carbon Neutrality, Sino-French Institute for Earth System Science, College of Urban and Environmental Sciences, Peking University, China; International Institute for Earth System Sciences, Nanjing University, China; Jiangsu Center for Collaborative Innovation in Geographic Information Resource Development and Application, China; Institute of Carbon Neutrality, Sino-French Institute for Earth System Science, College of Urban and Environmental Sciences, Peking University, China; School of Atmospheric Sciences, Guangdong Province Key Laboratory for Climate Change and Natural Disaster Studies, Key Laboratory of Tropical Atmosphere-Ocean System (Ministry of Education), Sun Yat-sen University, China; Institute of Carbon Neutrality, Sino-French Institute for Earth System Science, College of Urban and Environmental Sciences, Peking University, China; Department of Soil and Environment, Swedish University of Agricultural Sciences, Sweden; Institute of Carbon Neutrality, Sino-French Institute for Earth System Science, College of Urban and Environmental Sciences, Peking University, China; Jiangsu Key Laboratory of Atmospheric Environment Monitoring and Pollution Control, Collaborative Innovation Center of Atmospheric Environment and Equipment Technology, School of Environmental Science and Engineering, Nanjing University of Information Science & Technology (NUIST), China; Carbon-Water Research Station in Karst Regions of Northern Guangdong, School of Geography and Planning, Sun Yat-sen University, China; College of Meteorology and Oceanography, National University of Defense Technology, China; College of Geography and Remote Sensing, Hohai University, China

The terrestrial ecosystem plays an important role in regulating the regional carbon balance, in particular for China, where ecological projects during past decades [1,2] have led to large increases in China's land carbon sinks, partially offsetting fossil fuel emissions. Therefore, accurate evaluation of land carbon fluxes in China is critical for improving our understanding of the magnitude of China's carbon budget, and projecting its future changes. However, there are still large uncertainties in estimates of terrestrial carbon sinks in China based on inventory, eddy covariance, process-based carbon cycle modeling, and atmospheric inversion methods [3]. Carbon cycle models are not only applicable to estimate terrestrial carbon sinks and to project their future trends, but also can quantify the contribution of different drivers to changes in the land carbon sink [3]. The ensemble of estimates from multiple models is able to constrain the uncertainty in the estimated land carbon sink. So, this approach is used by the Global Carbon Budget (GCB) to estimate the global land carbon sink.

Although the GCB's model intercomparison project (trends and drivers of the regional scale terrestrial sources and sinks of carbon dioxide, or TRENDY) covers China spatially [4], simulation of China's land carbon cycle has some known limitations. First, the TRENDY project is driven by the LUH2 (Land-Use Harmonization) data [5], which cannot reflect historical land-use and land-cover change in China [6], and in particular cannot reproduce the rapid forest expansion since 1980 [2]. Second, the spatial resolution of the simulations in the TRENDY project is 0.5° × 0.5°, which is too coarse to assess finer-scale carbon sources and sinks in China (e.g. provinces or counties). Finally, these estimates did not fully consider carbon cycle processes such as lateral carbon transport, which resulted in potential biases [7]. In this study, we present a China Land Carbon Budget (CLCB) project, which is an open inter-model comparison project providing comprehensive estimates of the land carbon budget in China.

In this version (v1.0) of the CLCB, we solicited six carbon cycle models to voluntarily participate in the project (Supplementary S2.1) to provide estimates of China's land carbon sink with a spatial resolution of 0.1° × 0.1°. These models are widely used and have been at least partially trained and validated over China. All the models were forced with the same forcing (Supplementary Datasets) and following the same experimental protocol (S2.2 and Table S1) to estimate net biome production (NBP) considering effects of changes in climate, atmospheric CO_2_ and land-use and land-cover. Compared with the GCB, this study used a new attribution analysis method of land carbon sink change (S2.2 and Table S2). Unlike the global carbon cycle model ensemble that used a model product for land-use change [4], we applied a multiple-data fused land-use and land-cover dataset [8] to more realistically capture the rapid expansion of China's forests since 1980 (S1.2). In addition, a land-surface model that can simulate lateral organic carbon transport and a satellite-based wildfire inventory method (S2.3 and S2.4) were also used to constrain carbon cycle processes not included in the carbon cycle models. In the rest of the manuscript, we outline some of the key characteristics of CLCB v1.0 and the results of the model ensemble from 1980 to 2023.

As Fig. 1a shows, mean NBP simulated by the six models over the last 10 years (i.e. 2014–2023) was 0.327 ± 0.052 PgC yr^−1^. Soil organic carbon was transported laterally to the ocean and to other countries at the rate of 0.016 ± 0.001 PgC yr^−1^ and 0.007 ± 0.001 PgC yr^−1^, respectively (Fig. 1b). In addition, CO_2_ emissions from wildfires (fFire) were 0.021 ± 0.007 PgC yr^−1^ over the last 10 years (Fig. 1b). Consequently, after subtracting CO_2_ emissions and losses from wildfires and lateral carbon transport, the land carbon sink in China was 0.284 ± 0.055 PgC yr^−1^ (Fig. 1a). In contrast, the NBP estimated by the TRENDY project [5] showed a lower land carbon sink in China mainly because the land-use change dataset used by the TRENDY project has shown a decreased forest area since 1980 [2] (Figs S1–S3, S2.6). When compared with the national greenhouse gas inventory (NGHGI, S1.4), which provided the land carbon sink estimates for 1994, 2005, 2010, 2012, 2014, 2017 and 2018 (Fig. 1a), the estimates derived from NGHGI in 2014, 2017 and 2018 were comparable to those by this study in terms of estimate boundary (S1.4). The results showed the mean magnitude of land carbon sink over these three years is 0.319 ± 0.030 PgC yr^−1^ derived from NGHGI, which is quite close to our estimates (i.e. NBP-fFire, 0.317 ± 0.075 PgC yr^−1^).

**Figure 1.**
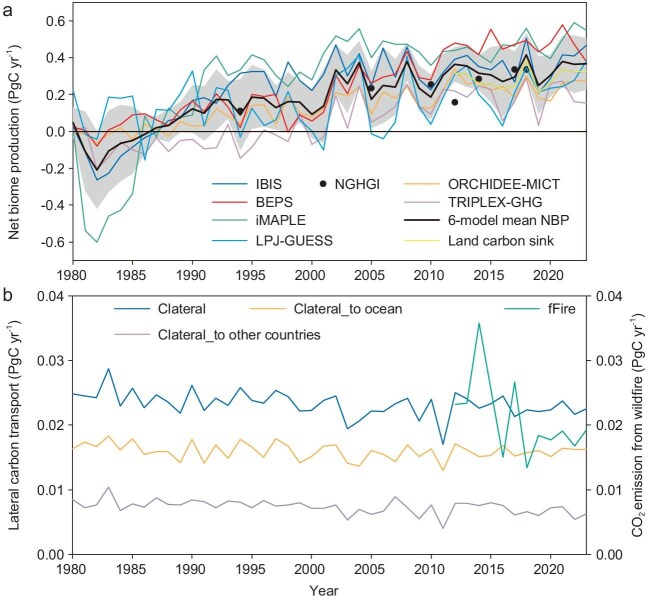
Terrestrial net biome production (NBP) in China as simulated by the BEPS, IBIS, iMAPLE, LPJ-GUESS, ORCHIDEE-MICT, and TRIPLEX-GHG models, as well as the multi-model average NBP (black line) with ±1 standard deviation (grey shaded area) (a). The land carbon sink from 2012 to 2023 (a) was calculated by subtracting lateral organic carbon transport (Clateral, b) and CO_2_ emissions from wildfires (fFire, b) from the NBP. Lateral organic carbon transport included carbon losses to the ocean and to other countries (b). The black dots in (a) refer to the reference values of the CO_2_ sink from the national greenhouse gas inventory (NGHGI).

From 1980 to 2023, the simulated NBP showed a substantial increment from −0.037 ± 0.088 PgC yr^−1^ in the 1980s to 0.327 ± 0.052 PgC yr^−1^ during the most recent 10-year period (Fig. 1a). Carbon emissions resulting from wildfires showed a marginally significant decreasing trend (−0.001 PgC yr^−2^, *p* = 0.07) from 2012 to 2023 (Fig. 1b). Total lateral carbon transport shows a significant decreasing trend (−0.00006 PgC yr^−2^, *p* < 0.01) since 1980 (Fig. 1b). Lateral transport of POC (particulate organic carbon) significantly decreased at −0.00007 PgC yr^−2^ (*p* < 0.01, Fig. S4a), and DOC (dissolved organic carbon) increased at a rate of 0.00001 PgC yr^−2^ (*p* = 0.42, Fig. S4b).

Our results indicate that China is among the countries showing the highest rates of increase in land carbon sinks (Table S3), which is about four times the global mean (Fig. S5). When isolating the contributions of land-use change, rising atmospheric CO_2_ concentration, and climate change to changes in land carbon sinks by the carbon cycle models and a bookkeeping model (LUCE) for land-use change (S2.2, S2.5 and Table S2), we found strong positive impacts of land-use change (E_LUC_) during the past four decades, enhancing China's land carbon sink by 0.100 ± 0.119 PgC yr^−1^ over this period (Fig. S6a), which is still slightly less than the positive impact of rising atmospheric CO_2_ concentration (0.147 ± 0.043 PgC yr^−1^) (Fig. S6b). The E_LUC_ estimated by the TRENDY project was lower than the estimates of the carbon cycle models and the LUCE model in this study (Fig. S6a), which is mainly due to different land-use change datasets (S2.6). The strong positive E_LUC_ in China was supported by a previous study [2]. In addition, we found that the positive E_LUC_ has been increasing since 1990 and has exceeded the contribution of CO_2_ since 2014 (Fig. S7). This is because the forest ecological projects from 1980 created vast areas of young and middle-aged forests, which are gradually entering a rapid growth stage and have considerable carbon sink capacity. On the contrary, climate change has reduced the national land carbon sink (−0.064 ± 0.054 PgC yr^−1^, Fig. S6c), but according to the NBP, in relatively cold regions such as the Tibetan Plateau and the northeastern and northwestern regions of China (Fig. S8d), the impacts of climate change remain positive. The attribution analysis results based on our method and the GCB's method were very similar in China (S2.2, Figs S9–S11, and Table S2).

Overall, CLCB v1.0 is an open multi-model ensemble platform that provides a fast-track assessment of China's land carbon sink, as well as the contributions by different factors. The magnitude of the land carbon sink by CLCB assessment is broadly consistent with estimates by previous data-driven models, process-based models, and atmospheric inversions (e.g. NGHGI, Table S4, [2,3,9,10]), but provides an uncertainty estimate considering model structures and uncertainties. Looking forward, the CLCB will continue to provide annual land carbon sink updates for China, involve more carbon cycle models, and provide data benchmarks and skill-weighted ensembles.

## Supplementary Material

nwaf052_Supplemental_File

## Data Availability

China's land carbon sink datasets are available through the project website (https://carbon.pku.edu.cn/data/English/index.htm).

## References

[1] Lu F, Hu H, Sun W et al. Proc Natl Acad Sci USA 2018; 115: 4039–44. 10.1073/pnas.1700294115 29666317 PMC5910802

[2] Yu Z, Ciais P, Piao S et al. Nat Commun 2022; 13: 5374. 10.1038/s41467-022-32961-2 36100606 PMC9470586

[3] Piao S, He Y, Wang X et al. Sci China Earth Sci 2022; 65: 641–51. 10.1007/s11430-021-9892-6

[4] Sitch S, O'Sullivan M, Robertson E et al. Glob Biogeochem Cycle 2024; 38: e2024GB008102. 10.1029/2024GB008102

[5] Friedlingstein P, O'Sullivan M, Jones MW et al. Earth Syst Sci Data 2023; 15: 5301–69. 10.5194/essd-15-5301-2023

[6] Wang X, Gao Y, Jeong S et al. Glob Biogeochem Cycle 2024; 38: e2023GB007865. 10.1029/2023GB007865

[7] Canadell JG, Monteiro PMS, Costa MH et al. Cambridge and New York: Cambridge University Press, 2021, 673–816.

[8] Xia X, Xia J, Chen X et al. J Geophys Res: Biogeosci 2023; 128: e2022JG007101. 10.1029/2022JG007101

[9] Wang Y, Wang X, Wang K et al. Nature 2022; 603: E7–9. 10.1038/s41586-021-04255-y 35296850

[10] Xia X, Ren P, Wang X et al. Sci Bull 2024; 69: 114–24. 10.1016/j.scib.2023.11.016 37989675

