# Supplementary material for: China Land Carbon Budget (CLCB1.0): a comprehensive estimate of the land carbon budget in China
Source: Natl Sci Rev. 2025 Feb 19;12(4):nwaf052. doi: 10.1093/nsr/nwaf052 (PMC11921772; doi:10.1093/nsr/nwaf052)
Supplement: nwaf052_Supplemental_File [file nwaf052_supplemental_file.docx]

***Supplementary Information for***

**China Land Carbon Budget (CLCB1.0): A comprehensive estimate of the land carbon budget in China**

Jiangzhou Xia^1^, Xiaosheng Xia^2^, Xuhui Wang^3*^, Weimin Ju^4,5*^, Zhengyang Lin^3^, Zhangcai Qin^6^, Yuxing Sang^3^, Yanzi Yan^7^, Wenping Yuan^3^, Xu Yue^8^, Haicheng Zhang^9^, Hao Zhou^10^ and Qiuan Zhu^11^

^1^Tianjin Key Laboratory of Water Resources and Environment, Tianjin Normal University, China; ^2^School of Atmospheric Sciences, Sun Yat-sen University, China; ^3^Institute of Carbon Neutrality, Sino-French Institute for Earth System Science, College of Urban and Environmental Sciences, Peking University, China; ^4^International Institute for Earth System Sciences, Nanjing University, China; ^5^Jiangsu Center for Collaborative Innovation in Geographic Information Resource Development and Application, China; ^6^School of Atmospheric Sciences, Guangdong Province Key Laboratory for Climate Change and Natural Disaster Studies, Key Laboratory of Tropical Atmosphere-Ocean System (Ministry of Education), Sun Yat-sen University, China; ^7^Department of Soil and Environment, Swedish University of Agricultural Sciences, Uppsala, Sweden; ^8^Jiangsu Key Laboratory of Atmospheric Environment Monitoring and Pollution Control, Collaborative Innovation Center of Atmospheric Environment and Equipment Technology, School of Environmental Science and Engineering, Nanjing University of Information Science & Technology (NUIST), China; ^9^Carbon-Water Research Station in Karst Regions of Northern Guangdong, School of Geography and Planning, Sun Yat-Sen University, China; ^10^College of Meteorology and Oceanography, National University of Defense Technology, China; ^11^College of Geography and Remote Sensing, Hohai University, China

^∗^Corresponding authors. E-mails: xuhui.wang@pku.edu.cn; juweimin@nju.edu.cn

**Supporting Text**

[1. Datasets 5](#_Toc188354080)

[1.1 Climate dataset 5](#_Toc188354081)

[1.2 Land-use and land-cover change (LUCC) dataset 5](#_Toc188354082)

[1.3 CO_2_ dataset 6](#_Toc188354083)

[1.4 National greenhouse gas inventory (NGHGI) 6](#_Toc188354084)

[2. Methods 7](#_Toc188354085)

[2.1 Models 7](#_Toc188354086)

[2.1.1 BEPS 7](#_Toc188354087)

[2.1.2 IBIS 8](#_Toc188354088)

[2.1.3 iMAPLE 9](#_Toc188354089)

[2.1.4 LPJ-GUESS 9](#_Toc188354090)

[2.1.5 ORCHIDEE-MICT 10](#_Toc188354091)

[2.1.6 TRIPLEX-GHG 10](#_Toc188354092)

[2.2 Simulation protocol 10](#_Toc188354093)

[2.3 Lateral organic carbon transport 12](#_Toc188354094)

[2.4 CO_2_ emissions from wildfires 13](#_Toc188354095)

[2.5 Bookkeeping model 13](#_Toc188354096)

[2.6 Comparison of China’s land carbon budget estimated in this study with the TRENDY project 14](#_Toc188354097)

[Figure S1. Temporal variation in simulated multi-model average net biome production (NBP) in China from 1980 to 2022 (a). NBP_ CLCB_ S3 refers to the NBP of the S3 experiment in this study. NBP_ GCB_ S3 refers to the NBP based on the S3 experiments of TRENDY project in the Global Carbon Budget 2023. 17](#_Toc188354098)

[Figure S2. Spatial pattern of multi-model average net biome production (NBP) in China and their differences from 1980 to 2022. NBP_CLCB_S3 refers to NBP of the S3 experiment in this study (a). NBP_GCB_S3 refers to the NBP based on the S3 experiments of TRENDY project in the Global Carbon Budget 2023 (b). NBP_CLCB_S3-NBP_GCB_S3 refers to the differences of NBP between NBP_CLCB_S3 and NBP_GCB_S3 (c). (d) refers to the change of forest fraction from 1980 to 2022 based on the land-use change data in this study. Data from Hong Kong, Macau, and Taiwan of China are not available in this study. 18](#_Toc188354099)

[Figure S3. Temporal changes of forest area in China from 1980 to 2022 based on the datasets in this study and LUH2 (Land-Use Harmonization). The LUH2 data was used by models of TRENDY project in the Global Carbon Budget 2023. 19](#_Toc188354100)

[Figure S4. Temporal variations in the lateral transport of particulate organic carbon (POC, a) and dissolved organic carbon (DOC, b). Lateral organic carbon (POC and DOC) transport includes carbon going to the ocean and to other countries. 20](#_Toc188354101)

[Figure S5. Temporal variation in simulated carbon sink density in China and global carbon sink density from 1980 to 2022. The net biome production (NBP) of the S3 experiment in China is from this study. The global mean value is calculated from the NBP of the S3 experiment of the Global Carbon Budget 2023. 21](#_Toc188354102)

[Figure S6. Contributions of land-use change (a, E_LUC_ effect), increasing atmospheric CO_2_ concentration (b, CO_2_ effect), and climate change (c, Climate effect) to terrestrial carbon sinks (i.e., NBP) as estimated by individual carbon models (BEPS, IBIS, iMAPLE, LPJ-GUESS, ORCHIDEE-MICT, and TRIPLEX-GHG models), as well as multi-model mean values (black line) with ±1 standard deviation (grey shaded area). GCB-2023-bookkeeping in (a) refers to the mean values of E_LUC_ based on the three bookkeeping models of Global Carbon Budget 2023. LUCE in (a) refers to the E_LUC_ simulated by the bookkeeping model (i.e., the LUCE model) [56]. GCB-2023-DGVMs refers to the multi-model mean values of attribution analysis based on twenty models in TRENDY project of the Global Carbon Budget 2023. Positive values indicate carbon sink effect. 22](#_Toc188354103)

[Figure S7. Contributions of land-use change (E_LUC_ effect), increasing atmospheric CO_2_ concentration (CO_2_ effect), and climate change (Climate effect) to terrestrial carbon sinks (i.e., NBP) as estimated by multi-model mean values of the six carbon models (BEPS, IBIS, iMAPLE, LPJ-GUESS, ORCHIDEE-MICT, and TRIPLEX-GHG models). Positive values indicate carbon sink effect. 23](#_Toc188354104)

[Figure S8. Spatial pattern of multi-model average net biome production (NBP) from 2014 to 2023 (a). Contributions of land-use change (b, E_LUC_ effect), increasing atmospheric CO_2_ concentration (c, CO_2_ effect), and climate change (d, Climate effect) to terrestrial carbon sinks (i.e., NBP). Data from Hong Kong, Macau, and Taiwan of China are not available in this study. 24](#_Toc188354105)

[Figure S9. Impact of different methods on the attribution analysis of changes in terrestrial carbon sinks. S0–S5 refer to the six modelling experiments (Section 2.2 and Table S2). Climate (a), CO_2_ (b), and E_LUC_ (c) effects refer to the contributions of climate change, increasing atmospheric CO_2_ concentration and land-use change to terrestrial carbon sinks (i.e., NBP). 25](#_Toc188354106)

[Figure S10. Contributions of land-use change (E_LUC_ effect), increasing atmospheric CO_2_ concentration (CO_2_ effect), and climate change (Climate effect) to terrestrial carbon sinks (i.e., NBP) based on IBIS model. The left panels (a, c, e) are based on the new method in this study. The right panels (b, d, f) are based on the Global Carbon Budget (GCB) method. Data from Hong Kong, Macau, and Taiwan of China are not available in this study. 26](#_Toc188354107)

[Figure S11. Contributions of land-use change (E_LUC_ effect), increasing atmospheric CO_2_ concentration (CO_2_ effect), and climate change (Climate effect) to terrestrial carbon sinks (i.e., NBP) based on ORCHIDEE-MICT model. The left panels (a, c, e) are based on the new method in this study. The right panels (b, d, f) are based on the Global Carbon Budget (GCB) method. Data from Hong Kong, Macau, and Taiwan of China are not available in this study. 27](#_Toc188354108)

[Table S1. Model experiments used in this study 28](#_Toc188354109)

[Table S2. Attribution analysis methods of terrestrial carbon sink change^a^ 29](#_Toc188354110)

[Table S3. Trends in national carbon sinks, 1980–2022^a^ 30](#_Toc188354111)

[Table S4. Comparison of previous terrestrial carbon sink estimates in China with this study 35](#_Toc188354112)

[References 36](#_Toc188354113)

# Datasets

## Climate dataset

This study aimed to report the results of the China Land Carbon Budget (CLCB1.0), which is part of the China greenhouse gas budget (CNGHG). To drive several ecosystem models for estimating CO_2_, CH_4_ and N_2_O sources and sinks, we produced a long-term climate forcing dataset from 1901 to 2023 by merging the CRU-JRA 2.4.5 [1-4] and ERA5-Land reanalysis datasets [5]. The detailed methods were introduced by Yuan et al. [6]. Briefly, the climate-forcing dataset included 13 meteorological variables with a spatial and temporal resolution of 0.1º×0.1º and 6-hour coverage from 1901 to 2023.

## Land-use and land-cover change (LUCC) dataset

The annual LUCC dataset from 1980 to 2023 was produced by fusing the Chinese Forest Cover Dataset (CFCD) and the China Land-Cover Data (CLCD) [7, 8]. This LUCC dataset did not include Taiwan Province due to lack of forest inventory data [9]. The 0.1º×0.1º LUCC dataset provided the proportions of the 10 land-use types and the transfer matrix between each pair of land-use types. The 10 land-use types included evergreen needleleaf forest, evergreen broadleaf forest, deciduous needleleaf forest, deciduous broadleaf forest, shrubland, C3 grass, C4 grass, C3 crops, C4 crops, and bare ground. Each model group converted the 10 land-use types to its own land-use types. From 1901 to 1959, harmonized land-use change data (LUH2) [10] were used to drive the process-based ecosystem models. Given the mismatch between LUH2 and our land-cover dataset as followed by Xia et al. [9], we took the simulation from 1960 to 1979 as a transition between the two LUCC datasets, and our land-cover dataset for 1980 was recycled for this period.

## CO_2_ dataset

The globally averaged annual atmospheric CO_2_ concentration over marine surface sites was provided by the US National Oceanic and Atmospheric Administration (NOAA) Global Monitoring Laboratory (<https://gml.noaa.gov/ccgg/trends/gl_data.html>).

## National greenhouse gas inventory (NGHGI)

In accordance with the relevant requirements of the United Nations Framework Convention on Climate Change (hereinafter referred to as “UNFCCC”), China has submitted the fourth National Communication (NC) on climate change of the People’s Republic of China and the third Biennial Update Report (BUR) on climate change of the People’s Republic of China to the secretariat of the UNFCCC, and reported to the international community on China’s information on China’s policies and actions to address climate change [11, 12]. The national greenhouse gas inventory (NGHGI) of China is reported in those two kinds of reports. The NGHGI used IPCC guidelines to estimate the China’s land carbon budget.

Till 2024, China has reported seven national greenhouse gas inventories, in 1994, 2005, 2010, 2012, 2014, 2017 and 2018 [11-16]. The land carbon budget in 2005 was first reported in the second NC and recalculated in the third BUR using updated method [12, 17]. We adopted the recalculated land carbon budget for the year 2005 in this study. For 2005, 2010, 2014, 2017, and 2018, the NGHGI covered six land use types: forest, cropland, grassland, wetland, construction land and other land. The land carbon budgets in those NGHGI were estimated as the changes of carbon stocks of aboveground biomass, underground biomass, litter, dead wood and soil organic carbon for each type of land. For 1994 and 2012, NGHGI’s land carbon budget estimates were not fully consistent in methodology and scope with the other five years of the inventory [14]. The land carbon budget estimates in NGHGI for 1994 and 2012 include changes in carbon stocks of biomass of forest and other woody plants, and the carbon emissions from conversion of forests to other land use types. However, NGHGI still covers the biomass carbon sink of forest and other woody plants, which is a major part of China’s land carbon sink [18]. The CO_2_ emissions from wildfires (fFire) was implicitly considered in NGHGI.

In order to maintain consistency in definitions of land carbon budgets between the NGHGI and this study, the fFire was subtracted from the net biome production (NBP) estimated in this study before comparing it to the NGHGI’s land carbon budget. In this study, we generated satellite-based fFire datasets between 2012 and 2023 (Section 2.4). We calculated the difference of NBP and fFire (NBP-fFire) from 2012 and 2023. The scope of NGHGI’s land carbon budget in 2012 was different from those in 2014, 2017 and 2018, so that year’s data was not included in the comparison. Finally, the land carbon budgets estimated by NGHGI and carbon cycle models in this study (i.e., NBP-fFire) were compared for 2014, 2017 and 2018.

# 2. Methods

## 2.1 Models

This study used multi-model ensemble mean values from simulations by the six process-based ecosystem models (BEPS, IBIS, iMAPLE, LPJ-GUESS, ORCHIDEE-MICT, and TRIPLEX-GHG) to indicate land carbon sinks (i.e., NBP) to avoid the simulation bias of any individual model. The following subsections contain detailed information for each model.

### 2.1.1 BEPS

The Boreal Ecosystem Productivity Simulator (BEPS) model is a diagnostic ecological model driven by remotely sensed vegetation parameters, including leaf area index (LAI), clumping index, and land-cover type, as well as meteorological and soil properties datasets [19]. There are daily and hourly versions of BEPS. This study used the daily version of BEPS to simulate photosynthesis, energy balance, and hydrological and soil biogeochemical processes. In the simulation of canopy gross primary productivity (GPP), the model used the leaf-level Farquhar’s biochemical model [20] with a two-leaf upscaling scheme from leaf to canopy [21]. Net primary productivity (NPP) was calculated as the residual of GPP minus autotrophic respiration, which was estimated according to biomass and temperature. Net ecosystem production (NEP) was quantified as the difference between NPP and heterotrophic respiration, which was simulated according to the Century model.

### 2.1.2 IBIS

The Integrated BIosphere Simulator (IBIS) was originally developed by Foley et al. [22] and was hierarchically organized to integrate various biophysical, physiological, and ecological processes of terrestrial ecosystems. A two-leaf Farquhar model was used by IBIS to simulate canopy photosynthesis [20], and the vegetation biomass of leaves, stems, and roots was simulated by the processes of net primary production allocation, mortality and tissue turnover [22]. Soil heterotrophic respiration was simulated based on changes in several carbon pools, including microbial, litter, and soil carbon pools. In the past few years, several key ecological processes of the IBIS model have been revised and developed to improve model simulation accuracy, including plant carbon allocation [23], plant phenology [24], dynamic root growth [25], wetland methane (CH_4_) emissions [26], and nitrous oxide (N_2_O) emissions [27]. The IBIS model has been extensively applied and validated for estimating terrestrial carbon sinks in China [9, 28, 29]. Since 2020, IBIS model participated in the multi-model intercomparison project (the TRENDY project) of the Global Carbon Budget (GCB), which provides estimates of the global terrestrial carbon budget annually.

### 2.1.3 iMAPLE

The interactive Model of Air Pollution and Land Ecosystems (iMAPLE) is the updated version of the Yale Interactive Terrestrial Biosphere Model (YIBs) [30] with dynamic coupling of terrestrial carbon and water cycles [31]. The iMAPLE applies the well-established Michaelis-Menten enzyme-kinetics scheme [20] for plant photosynthesis and two-leaf models [32] for canopy radiative transfer. The model can simulate vegetation growth and development through carbon allocation among leaves, stems, and roots. iMAPLE can also simulate terrestrial water processes including runoff and evapotranspiration [33], and implements hourly couplings of leaf photosynthesis and transpiration based on the Ball-Berry stomatal conductance scheme [34]. Furthermore, iMAPLE features unique biogeochemical processes, including process-based fire emissions, prognostic wetland methane emissions, and trait-based ozone vegetation damage [31]. In recent years (2020–2024), iMAPLE (referred to previously as YIBs) participated in the TRENDY project of the GCB to estimate both historical and present-day land carbon sink capacity at global scale.

### 2.1.4 LPJ-GUESS

The Lund-Potsdam-Jena General Ecosystem Simulator (LPJ-GUESS) is a process-based dynamic vegetation model that simulates carbon and nitrogen and is designed for regional to global applications [35]. Plant physiological processes simulated in LPJ-GUESS are individual plant-based, including establishment, growth, succession of cohorts (each represented by an average individual of a plant functional type or species), as well as competition for light, space, and soil resources. The latest version of LPJ-GUESS v4.1.1, which is available online (<https://zenodo.org/records/8065737>), was used in this study. It incorporates development and improvement of the terrestrial nitrogen cycle [35], crop management [36], and forest management [37]. The LPJ-GUESS model has participated in all versions of the TRENDY project, which was started in 2009 (TRENDY‐v1) [38].

### 2.1.5 ORCHIDEE-MICT

Organizing Carbon and Hydrology in Dynamic EcosystEms – aMeliorate Interactions between Carbon and Temperature (ORCHIDEE-MICT) was developed based on the ORCHIDEE model [39]. Compared with the trunk version, it considers more processes related to two carbon pools: permanently frozen soils (permafrost), and the great expanse of boreal forest in cold biomes and describes the interactions among soil carbon, soil temperature, and hydrology. It also incorporates new parameterizations for crop irrigation [40]. The model has been evaluated over the last two or three decades and shows good performance against empirically generated datasets [41]. The ORCHIDEE model and its branches participated in the TRENDY project from 2013 (TRENDY‐v2) [38].

### 2.1.6 TRIPLEX-GHG

TRIPLEX-GHG is a process-based dynamic global vegetation model. This model can simulate cycling of carbon, nitrogen, and phosphorus, greenhouse gas emissions (CO_2_, CH_4_, N_2_O), vegetation phenology and dynamics, soil biogeochemical processes, wetland area dynamics, the peatland carbon cycle, and LUCC [42-45]. The TRIPLEX-GHG model uses plant functional types (PFTs) to represent different vegetation. Canopy photosynthesis was modeled based on the Farquhar model to calculate GPP and NPP for each PFT [46]. By coupling the carbon and nitrogen cycles, the model can simulate the nitrogen limitation on canopy NPP [44, 47]. Eleven types of land conversions/disturbances were included to represent LUCC impacts on the carbon cycle, which were based on the framework of a previous study [48].

## 2.2 Simulation protocol

Six model experiments (S0–S5) were implemented to quantify the contributions of land-use change, rising atmospheric CO_2_ concentration, and climate change on the terrestrial carbon cycle in China (Table S1).

S0: control simulation. No forcing change, i.e., climate, the atmospheric CO_2_ concentration, and land-cover datasets in 1901 were used for 1901–2023.

S1: simulation with only CO_2_ temporally varied.

S2: simulation with only CO_2_ and climate temporally varied.

S3: simulation with CO_2_, climate and land use temporally varied.

S4: simulation with only CO_2_ and land use temporally varied.

S5: simulation with only climate and land use temporally varied.

To reach a steady state of the terrestrial ecosystem models, a spin-up run was needed before launching the model experiments described above. In spin-up period, we spun the model by recycling climate variables through 1901–1920, CO_2_ concentration as in 1901 (i.e., 296.8 ppm), and LUH2’s land-cover data in 1901. Following the method of GCB [49], the steady state was defined as mean carbon flux (i.e., NEP) over the latest 100 years < 0.03 Gt CO_2_ yr^–1^ and the change during the latest 100 years < 0.01 Gt CO_2_ yr^–1^ [9].

This study used experiments S3, S4, and S5 to quantify the contributions of climate change (S3–S4, i.e., the difference in net biome production (NBP) between the S3 and S4 experiments, Climate effect), rising atmospheric CO_2_ concentration (S3–S5, CO_2_ effect), and land-use change (S4+S5-S3, E_LUC_ effect) to the terrestrial carbon cycle (Table S2). Note that this is an improved model experimental scheme compared to the GCB [49], which quantifies the effects of climate, CO_2_, and land-use change based on S2–S1, S1–S0, and S3–S2 respectively (Table S2). The GCB’s experimental schemes use invariant LUCC when quantifying the impacts of climate change and rising atmospheric CO_2_ concentration, which omits the impact of LUCC. We ran two models (IBIS and ORCHIDEE-MICT) to compare the differences in the two experimental schemes by running all S0–S5 experiments. Basically, the results derived from the two experimental schemes were quite similar (Figs. S9, S10, and S11). However, because the S3–S5 experiments all accounted for actual land-use change, the effects of climate, CO_2_, and land-use change on terrestrial carbon sinks diagnosed by the new analysis method had larger inter-annual variability than the analysis method used for the GCB (Fig. S9 and Table S2). The spatial patterns of attribution analysis were very similar between these two approaches based on the IBIS (Fig. S10) and ORCHIDEE-MICT (Fig. S11) models, respectively.

Our simulation protocol and the factorial experimental designs helped us to isolate the effects of climate change, atmospheric CO_2_ increment, and land-use change. Note also that, given the model limitations in representing fire events and lateral organic carbon transport, which are critical for the land carbon budget, we separately considered carbon emissions from fire and lateral organic carbon transport, which were estimated using remote-sensing data and a land surface model.

## 2.3 Lateral organic carbon transport

Lateral organic carbon transport caused by soil erosion and leaching in China along the land-river-ocean continuum was simulated by the ORCHIDEE-C_lateral_ land surface model [50, 51]. ORCHIDEE-C_lateral_ simulates the vertical carbon fluxes among atmosphere, vegetation, and soil, the lateral fluxes of particulate and dissolved carbon from terrestrial ecosystems to the ocean through inland water networks, and the interactions between vertical and lateral carbon fluxes. This model has been evaluated against previous global-scale estimates using data-driven methods or mass-balance approaches, as well as measurements of riverine water discharge, sediment concentrations, particulate organic carbon (POC), and dissolved organic carbon (DOC) at hydrological gauging stations along large global rivers. In this study, the ORCHIDEE-C_lateral_ model was applied to simulate lateral carbon transport across China from 1901 to 2023. Based on the simulation results, we analyzed lateral carbon delivery from terrestrial ecosystems to the river networks, carbon deposition on riverbeds and floodplains, and carbon export from China to the seas and to neighboring countries.

## 2.4 CO_2_ emissions from wildfires

In this study, we generated data for CO_2_ emissions from wildfires between 2012 and 2023. To explore national wildfire-induced CO_2_ emissions, the study employed satellite-based data on burned vegetation to generate the China Wildfire Emissions Dataset (ChinaWED) [52]. This newly developed dataset adheres to a consistent calculation framework, incorporating updated components such as burned area, emission factors, and fuel loads. Burned area estimation relies on the MODIS burned-area product, supplemented by finer-resolution spatial data from VIIRS fire detections [53]. Emission factors were collected and summarized from previous research conducted in China and neighboring regions, encompassing diverse land-cover types and crops. Aboveground biomass products with higher resolution served as a proxy for fuel loadings [54].

## 2.5 Bookkeeping model

The Land-Use Change Emissions (LUCE) model is based on a bookkeeping approach to simulate land-use change related greenhouse gas emissions [55]. It features sub-grid gross land-use transitions and considers grid carbon density changes. The impact of land cohort age on secondary land carbon stocks is specifically considered, with particular emphasis on young secondary land involved in shifting cultivation practices. The model incorporates four major simulation processes to estimate emissions related to LUCC activities, including deforestation, afforestation and reforestation, wood harvest and subsequent regrowth, and other conversions between land uses. The four processes (clearing, abandonment, harvest, and others) can reflect different changes of carbon in vegetation, soil, and possibly wood products following an LUCC activity. With LUCE, both gross emissions (source or sink) and net emissions (E_LUC_) can be attributed to specific LUCC activities over time and space as needed [56]. The LUCE model was driven by the new LUCC dataset of this study (Datasets 1.2).

## 2.6 Comparison of China’s land carbon budget estimated in this study with the TRENDY project

There are some differences between the model configuration and driving forces of this study (CLCB v1.0) and TRENDY project (TRENDY-v12) of the Global Carbon Budget 2023 (GCB-2023). First, the spatial resolution of climate forcing of the TRENDY project is different with this study. The climate forcing of TRENDY project from 1901 to 2022 is CRU-JRA 2.4.5 [1-4] and a revised radiation dataset [57] with a spatial resolution of 0.5º×0.5º. The climate forcing of CLCB includes a revised CRU-JRA 2.4.5 (1901-1949, 0.1º×0.1º) and the original ERA5-Land (1950-2023, 0.1º×0.1º) reanalysis datasets [5] (see Section 1.1). Second, the TRENDY project is driven by the LUH2 dataset, which cannot reflect historical land-use and land-cover change in China [58]. We developed a LUCC dataset to more realistically capture the rapid expansion of forests since 1980 in China (see Section1.2). Third, this study used a new attribution analysis method of land carbon sink change (see Section 2.2 and Table S2). The attribution analysis results from our method and the method of GCB-2023 were quite similar in China (Figs. S9, S10, and S11). This means that we can compare the results of attribution analysis in this study with those of TRENDY project. Fourth, the wildfires emission (i.e., fFire) was considered in the most of models (13 out of 20 models) for simulating NBP in the simulation experiments of TRENDY project [49], which was not considered in the NBP simulation in this study.

In order to compare the impact of LUCC dataset on the estimation of land carbon sinks in China, we used the multi-model ensemble mean values of NBP from the S3 experiment of TRENDY project to indicate land carbon sink. This is consistent with the method in this study. Ideally, we should first subtract fFire from our NBP and then compare it to the NBP of TRENDY project. However, we only generated satellite-based fFire datasets between 2012 and 2023 (Section 2.4). Considering that fFire only accounts for 6% of the NBP (see Fig.1 in the main text) and is simulated by part of the TRENDY models in GCB-2023, we directly compare the spatial and temporal differences of NBP from 1980 to 2022 in this study with that of TRENDY models. The NBP datasets from twenty models of TRENDY project [49] were resampled to a spatial resolution of 0.1º×0.1º using nearest neighbor interpolation method.

We compared our NBP from the S3 experiment (hereafter NBP_CLCB_S3) with the NBP from S3 experiments of TRENDY models in GCB-2023 (hereafter NBP_GCB_S3). In terms of magnitude, NBP_GCB_S3 was lower than NBP_CLCB_S3 (Fig. S1). In terms of interannual variability, NBP_CLCB_S3 was well correlated with NBP_GCB_S3 (R^2^ = 0.77, Fig. S1). As for the spatial pattern, the NBP derived from NBP_CLCB_S3 was larger than those of NBP_GCB_S3 mainly in the forested region (Fig. S2). These differences were mainly due to the inability of the LUH2 land-use change dataset to capture the rapid expansion of forest in China since 1980 (Figs. S2d and S3) [9, 58].

The attribution analysis showed that the contribution of land-use change to land carbon sink (E_LUC_ effect) in China based on the TRENDY models of GCB-2023 (i.e., GCB-2023-DGVMs in Fig. S6a) was lower than that of this study (Fig. S6a). The mean values of E_LUC_ based on the three bookkeeping models (i.e., BLUE [59], OSCAR [60], and H&C2023 [61]) of GCB-2023 (i.e., GCB-2023-bookkeeping in Fig. S6a) also was lower than that of this study, but larger than the estimate of GCB-2023-DGVMs (Fig. S6a). One of the main reasons for these differences is that the three bookkeeping models used different land-use change datasets [49]. The BLUE model used the LUH2 dataset, which is same with the TRENDY models. The H&C2023 model used the data of FAO. The OSCAR model was run with both LUH2 and FAO datasets. When compared with the bookkeeping model (i.e., LUCE model, Section 2.5) [56], which was driven by our new LUCC dataset (Section 1.2), the LUCE model also simulated the strong positive E_LUC_ like the estimates of carbon cycle models in this study (Fig. S6a). Those results highlighted the importance of reliable LUCC dataset in land carbon budget estimation. In addition, the contribution of rising atmospheric CO_2_ concentration to changes in China’s land carbon sink based on TRENDY models was larger than that of this study (Fig. S6b). The impact of climate change on China’s land carbon sink based on TRENDY models was similar with that of this study (Fig. S6c). Except the LUCC dataset, the different models and climate forcing datasets may also lead to differences in attribution analysis of terrestrial carbon sink change [62]. These differences can be quantitatively analyzed through model experiments in future studies.


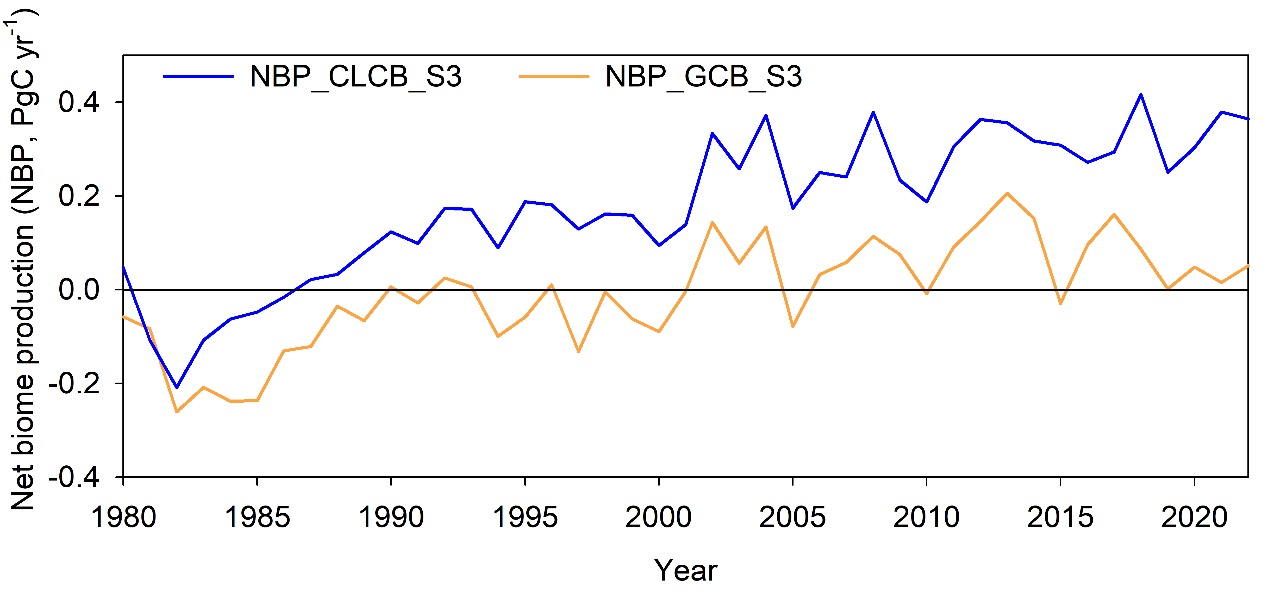


# Figure S1. Temporal variation in simulated multi-model average net biome production (NBP) in China from 1980 to 2022 (a). NBP_ CLCB_ S3 refers to the NBP of the S3 experiment in this study. NBP_ GCB_ S3 refers to the NBP based on the S3 experiments of TRENDY project in the Global Carbon Budget 2023.


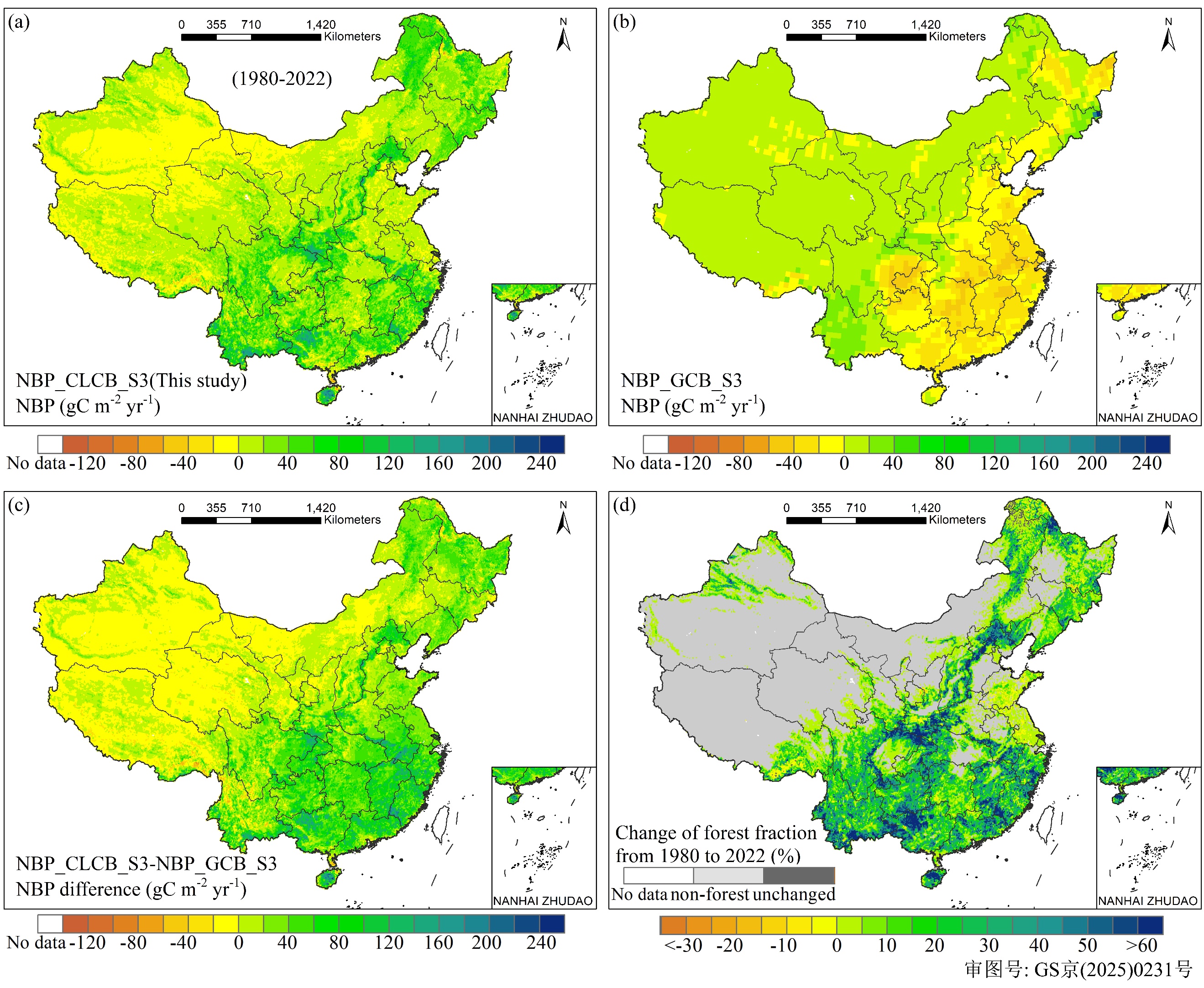


# Figure S2. Spatial pattern of multi-model average net biome production (NBP) in China and their differences from 1980 to 2022. NBP_CLCB_S3 refers to NBP of the S3 experiment in this study (a). NBP_GCB_S3 refers to the NBP based on the S3 experiments of TRENDY project in the Global Carbon Budget 2023 (b). NBP_CLCB_S3-NBP_GCB_S3 refers to the differences of NBP between NBP_CLCB_S3 and NBP_GCB_S3 (c). (d) refers to the change of forest fraction from 1980 to 2022 based on the land-use change data in this study. Data from Hong Kong, Macau, and Taiwan of China are not available in this study.


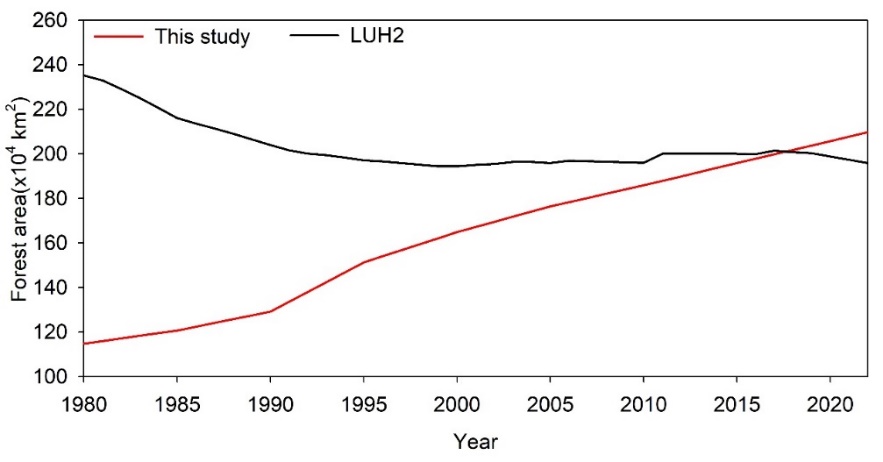


# Figure S3. Temporal changes of forest area in China from 1980 to 2022 based on the datasets in this study and LUH2 (Land-Use Harmonization). The LUH2 data was used by models of TRENDY project in the Global Carbon Budget 2023.


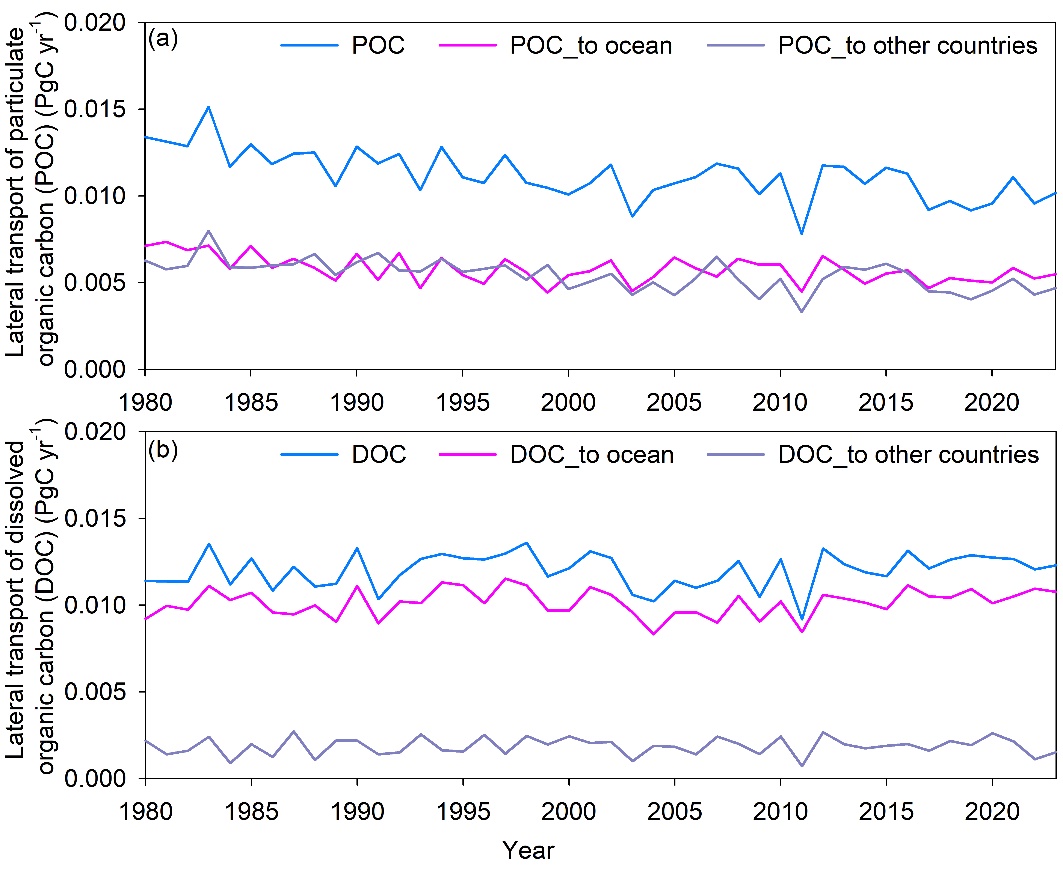


# Figure S4. Temporal variations in the lateral transport of particulate organic carbon (POC, a) and dissolved organic carbon (DOC, b). Lateral organic carbon (POC and DOC) transport includes carbon going to the ocean and to other countries.


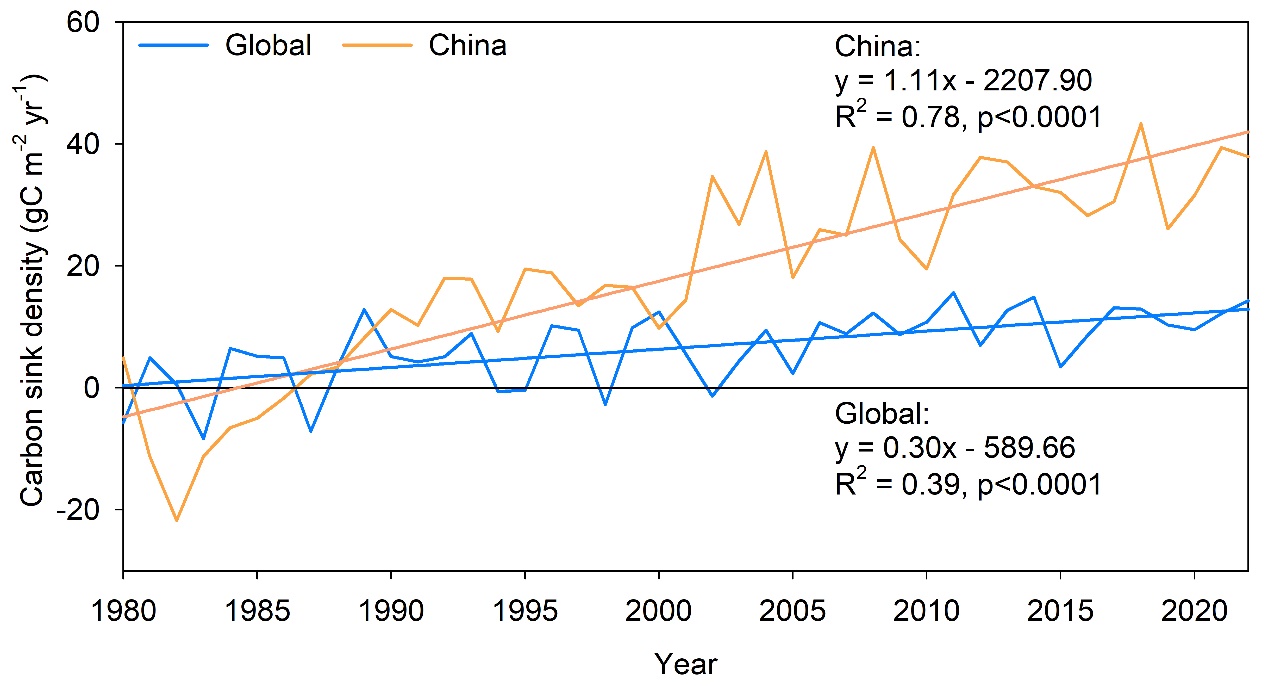


# Figure S5. Temporal variation in simulated carbon sink density in China and global carbon sink density from 1980 to 2022. The net biome production (NBP) of the S3 experiment in China is from this study. The global mean value is calculated from the NBP of the S3 experiment of the Global Carbon Budget 2023.


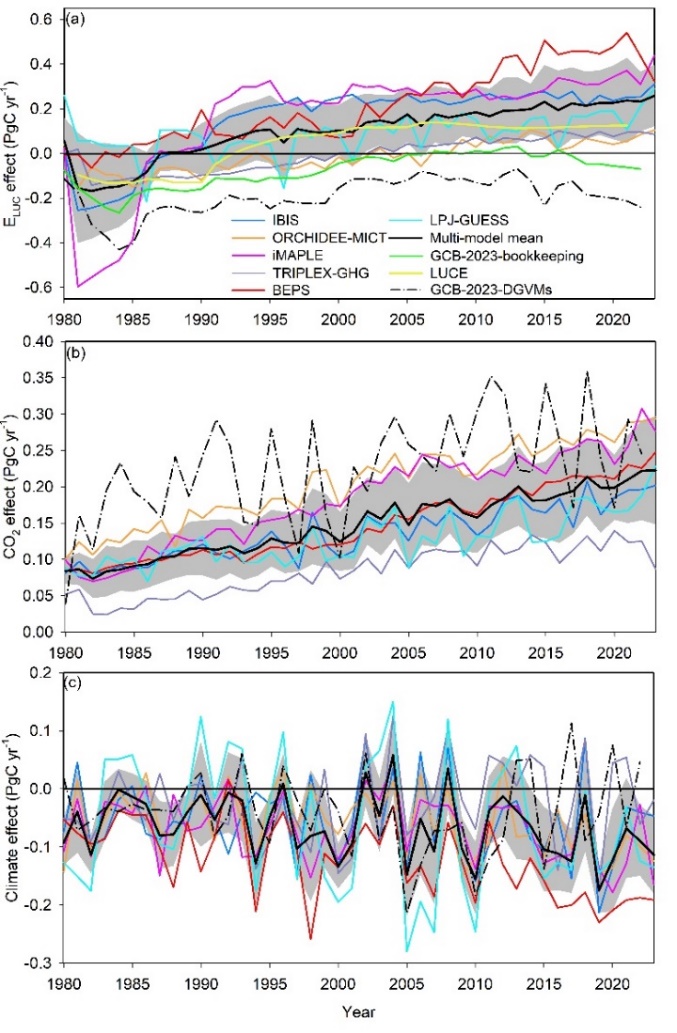


# Figure S6. Contributions of land-use change (a, E_LUC_ effect), increasing atmospheric CO_2_ concentration (b, CO_2_ effect), and climate change (c, Climate effect) to terrestrial carbon sinks (i.e., NBP) as estimated by individual carbon models (BEPS, IBIS, iMAPLE, LPJ-GUESS, ORCHIDEE-MICT, and TRIPLEX-GHG models), as well as multi-model mean values (black line) with ±1 standard deviation (grey shaded area). GCB-2023-bookkeeping in (a) refers to the mean values of E_LUC_ based on the three bookkeeping models of Global Carbon Budget 2023. LUCE in (a) refers to the E_LUC_ simulated by the bookkeeping model (i.e., the LUCE model) [56]. GCB-2023-DGVMs refers to the multi-model mean values of attribution analysis based on twenty models in TRENDY project of the Global Carbon Budget 2023. Positive values indicate carbon sink effect.


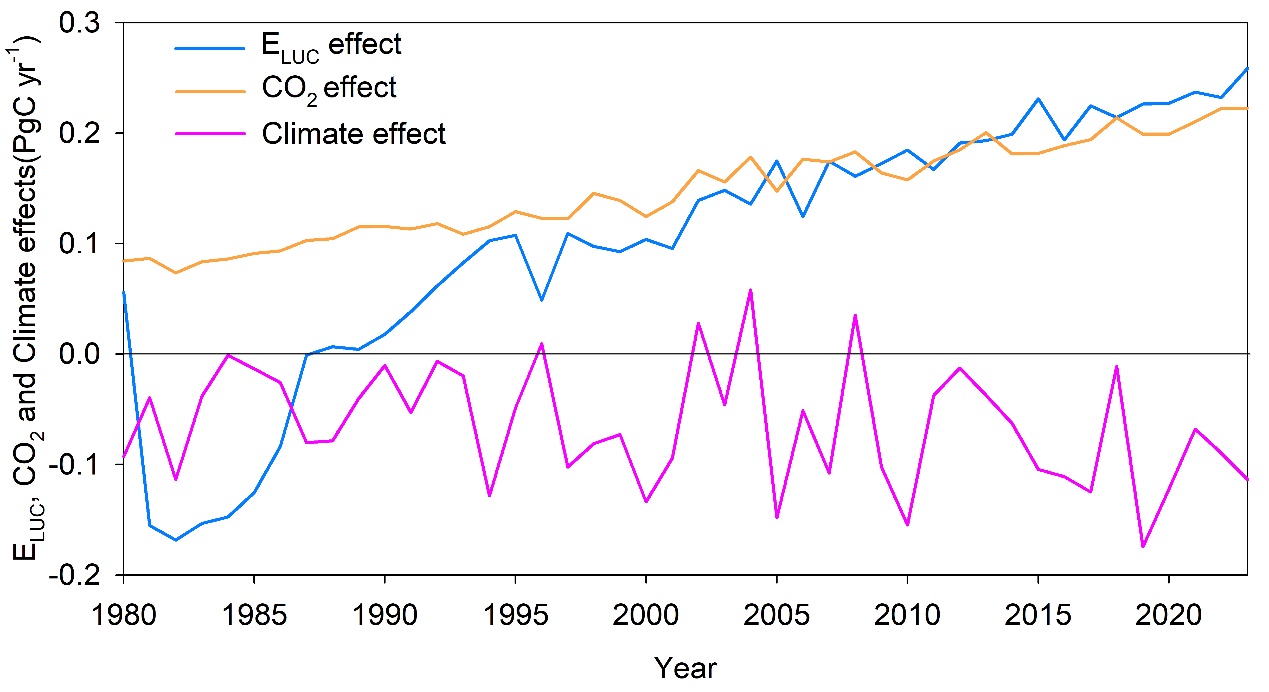


# Figure S7. Contributions of land-use change (E_LUC_ effect), increasing atmospheric CO_2_ concentration (CO_2_ effect), and climate change (Climate effect) to terrestrial carbon sinks (i.e., NBP) as estimated by multi-model mean values of the six carbon models (BEPS, IBIS, iMAPLE, LPJ-GUESS, ORCHIDEE-MICT, and TRIPLEX-GHG models). Positive values indicate carbon sink effect.


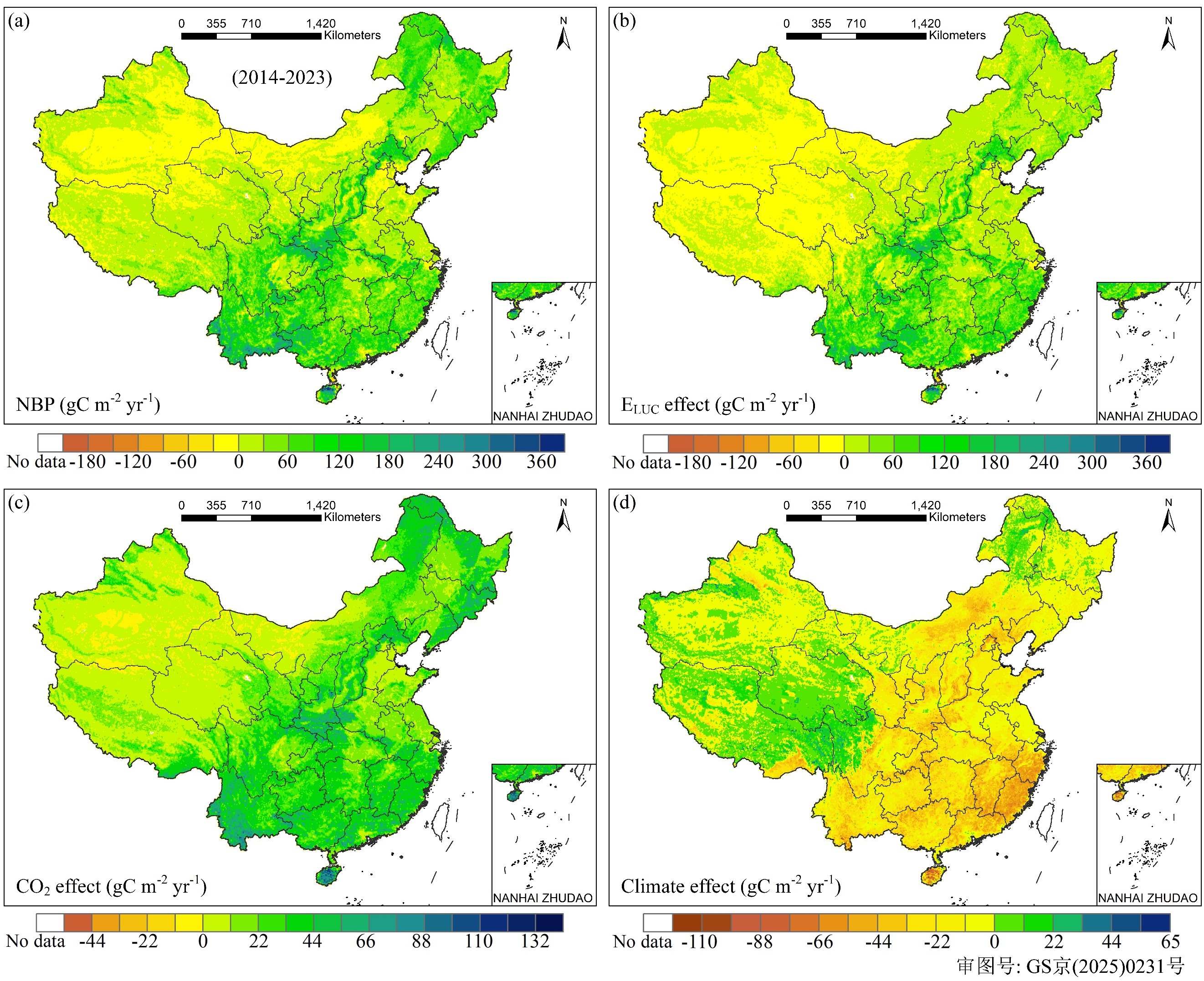


# Figure S8. Spatial pattern of multi-model average net biome production (NBP) from 2014 to 2023 (a). Contributions of land-use change (b, E_LUC_ effect), increasing atmospheric CO_2_ concentration (c, CO_2_ effect), and climate change (d, Climate effect) to terrestrial carbon sinks (i.e., NBP). Data from Hong Kong, Macau, and Taiwan of China are not available in this study.


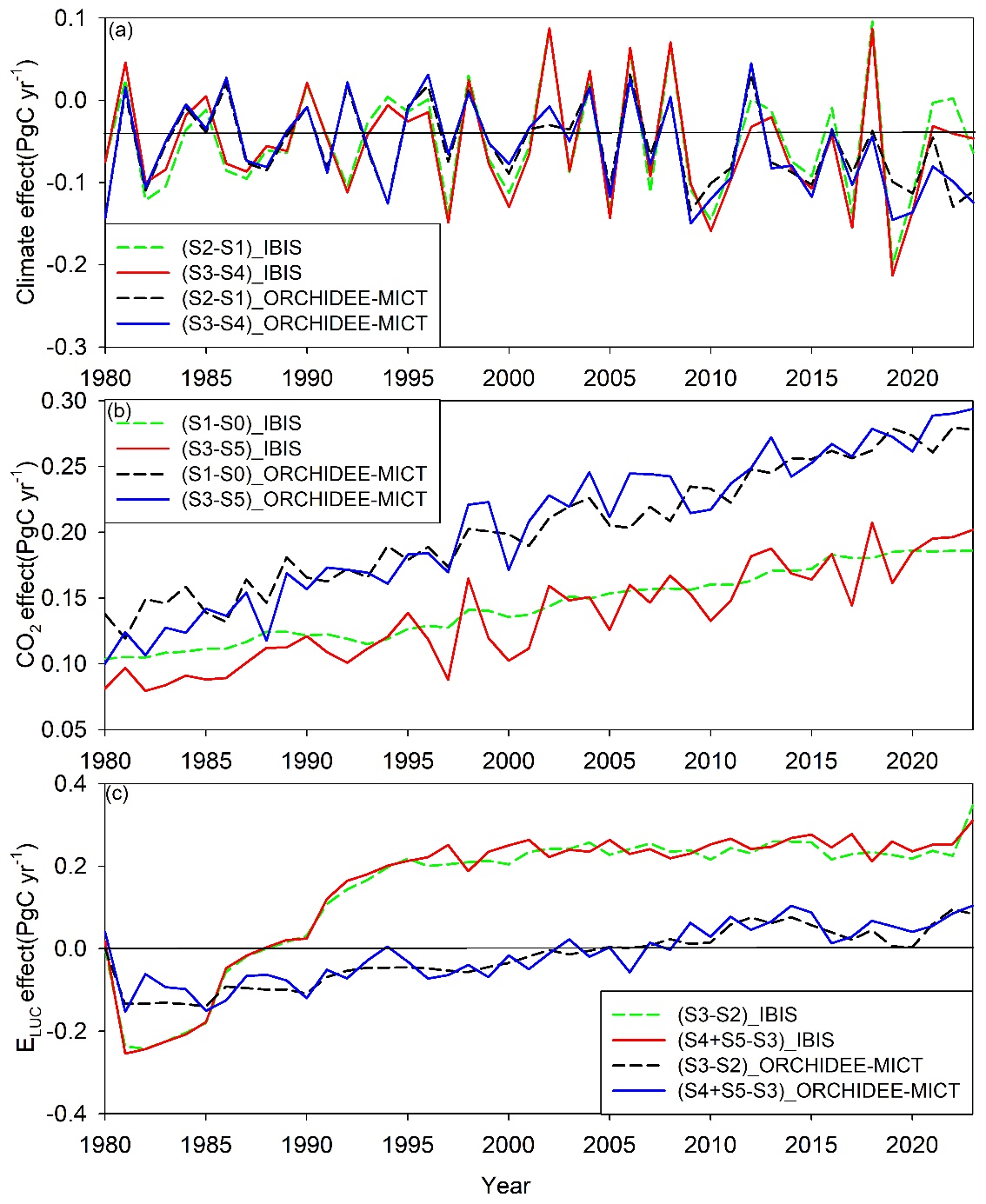


# Figure S9. Impact of different methods on the attribution analysis of changes in terrestrial carbon sinks. S0–S5 refer to the six modelling experiments (Section 2.2 and Table S2). Climate (a), CO_2_ (b), and E_LUC_ (c) effects refer to the contributions of climate change, increasing atmospheric CO_2_ concentration and land-use change to terrestrial carbon sinks (i.e., NBP).


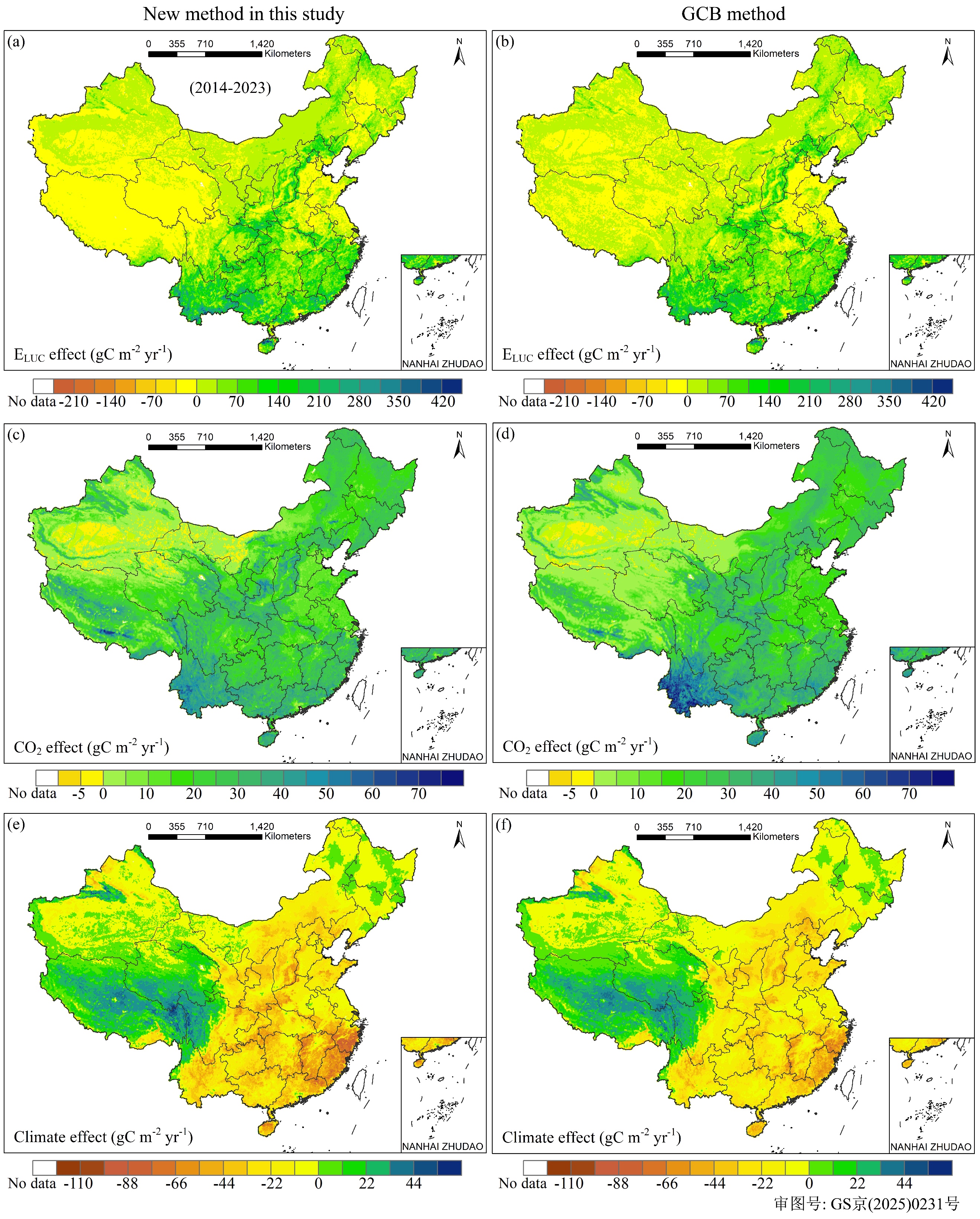


# Figure S10. Contributions of land-use change (E_LUC_ effect), increasing atmospheric CO_2_ concentration (CO_2_ effect), and climate change (Climate effect) to terrestrial carbon sinks (i.e., NBP) based on IBIS model. The left panels (a, c, e) are based on the new method in this study. The right panels (b, d, f) are based on the Global Carbon Budget (GCB) method. Data from Hong Kong, Macau, and Taiwan of China are not available in this study.


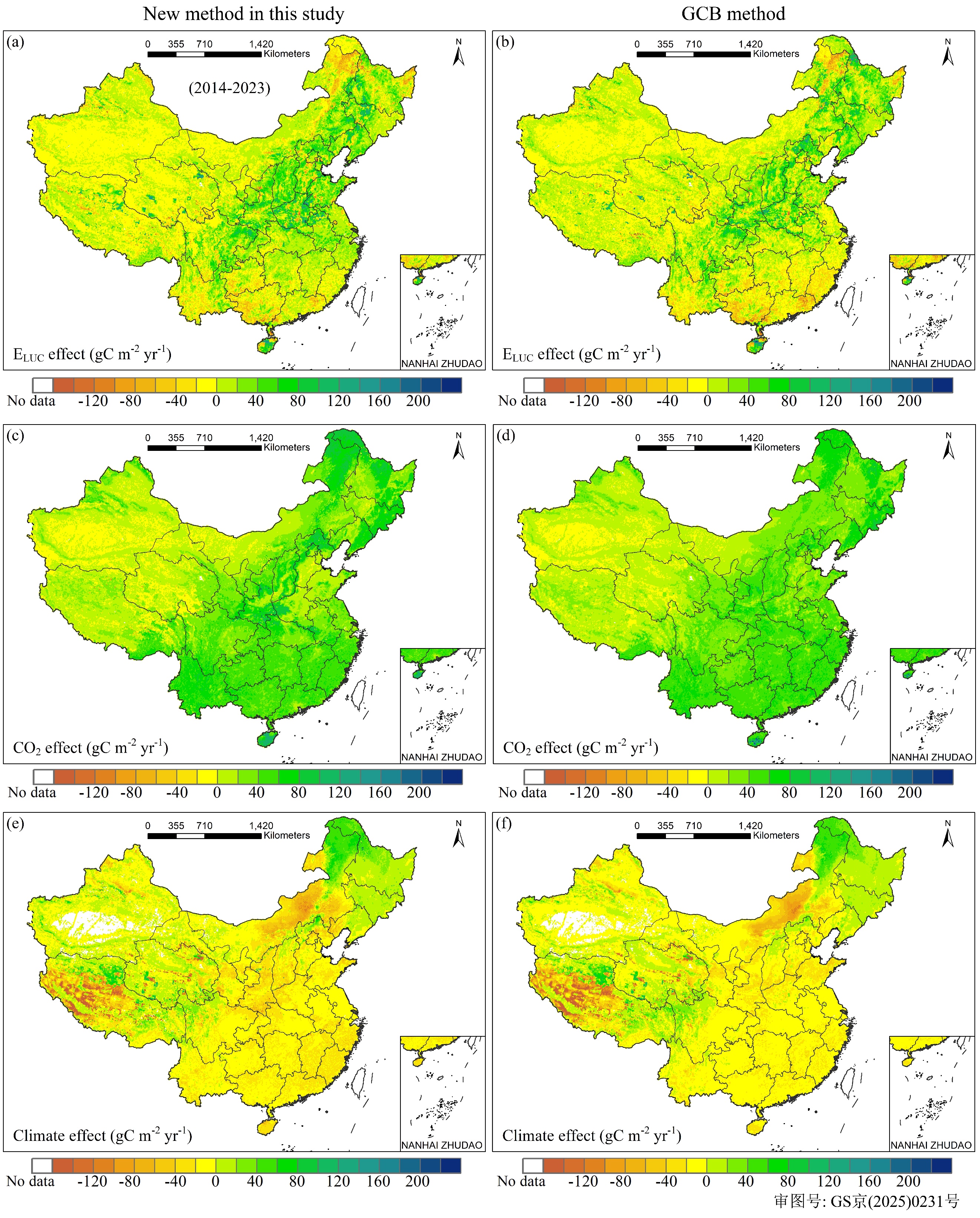


# Figure S11. Contributions of land-use change (E_LUC_ effect), increasing atmospheric CO_2_ concentration (CO_2_ effect), and climate change (Climate effect) to terrestrial carbon sinks (i.e., NBP) based on ORCHIDEE-MICT model. The left panels (a, c, e) are based on the new method in this study. The right panels (b, d, f) are based on the Global Carbon Budget (GCB) method. Data from Hong Kong, Macau, and Taiwan of China are not available in this study.

# Table S1. Model experiments used in this study

| Model experiment  code | Climate | CO_2_ | Land-use change |
| --- | --- | --- | --- |
| S0 | 1901 | 1901 | 1901 |
| S1 | 1901 | 1901−2023 | 1901 |
| S2 | 1901−2023 | 1901−2023 | 1901 |
| S3 | 1901−2023 | 1901−2023 | 1901−2023 |
| S4 | 1901 | 1901−2023 | 1901−2023 |
| S5 | 1901−2023 | 1901 | 1901−2023 |

# Table S2. Attribution analysis methods of terrestrial carbon sink change^a^

|  | Climate effect | CO_2_ effect | Land-use change (E_LUC_) effect |
| --- | --- | --- | --- |
| Global Carbon Budget (GCB) method | S2–S1 | S1–S0 | S3–S2 |
| New analysis method | S3–S4 | S3–S5 | S4+S5–S3 |

^a^The contribution of the Climate, CO_2_, and E_LUC_ effects to the terrestrial carbon sink can be distinguished by applying the equations in this table to the net biome production (NBP) of the different modelling experiments (S0–S5).

# Table S3. Trends in national carbon sinks, 1980–2022^a^

| **ID** | **Country/region name** | **Trend (TgC yr^-2^)** |
| --- | --- | --- |
| 1 | Afghanistan | -0.024631 |
| 2 | Albania | 0.011429 |
| 3 | Algeria | 0.028422 |
| 4 | Andorra | 0.000874 |
| 5 | Angola | 1.078400 |
| 6 | Antigua and Barbuda | -0.000021 |
| 7 | Argentina | -1.291000 |
| 8 | Armenia | -0.004832 |
| 9 | Australia | 1.449500 |
| 10 | Austria | 0.026183 |
| 11 | Azerbaijan | -0.029664 |
| 12 | Bahamas | 0.001030 |
| 13 | Bahrain | 0.000147 |
| 14 | Bangladesh | 0.035183 |
| 15 | Barbados | 0.000026 |
| 16 | Belarus | 0.122330 |
| 17 | Belgium | -0.008901 |
| 18 | Belize | 0.010631 |
| 19 | Benin | 0.021528 |
| 20 | Bhutan | 0.003533 |
| 21 | Bolivia | -0.313640 |
| 22 | Bosnia and Herzegovina | 0.017494 |
| 23 | Botswana | 0.508020 |
| 24 | Brazil | 4.192400 |
| 25 | Brunei Darussalam | 0.004093 |
| 26 | Bulgaria | 0.072164 |
| 27 | Burkina Faso | 0.157100 |
| 28 | Burundi | 0.007017 |
| 29 | Cambodia | 0.206330 |
| 30 | Cameroon | 0.325580 |
| 31 | Canada | 3.250300 |
| 32 | Cape Verde | 0.000056 |
| 33 | Central African Republic | 0.646920 |
| 34 | Chad | 0.426440 |
| 35 | Chile | -0.012495 |
| 36 | China | 10.696209 |
| 37 | Colombia | 0.714570 |
| 38 | Comoros | 0.000245 |
| 39 | Congo | 0.162290 |
| 40 | Cook Islands | 0.000013 |
| 41 | Costa Rica | 0.153450 |
| 42 | Côte d'Ivoire | 0.469510 |
| 43 | Croatia | 0.032184 |
| 44 | Cuba | 0.177930 |
| 45 | Cyprus | -0.000417 |
| 46 | Czechia | -0.024971 |
| 47 | Democratic Republic of the Congo | -0.382870 |
| 48 | Denmark | 0.002710 |
| 49 | Djibouti | -0.001964 |
| 50 | Dominica | 0.000130 |
| 51 | Dominican Republic | 0.071082 |
| 52 | Ecuador | 0.519100 |
| 53 | Egypt | 0.005365 |
| 54 | El Salvador | 0.030881 |
| 55 | Equatorial Guinea | 0.013647 |
| 56 | Eritrea | 0.061345 |
| 57 | Estonia | 0.009883 |
| 58 | Eswatini | 0.014884 |
| 59 | Ethiopia | 0.502850 |
| 60 | Fiji | 0.020552 |
| 61 | Finland | 0.059502 |
| 62 | France | -0.101820 |
| 63 | Gabon | 0.189340 |
| 64 | Gambia | 0.008267 |
| 65 | Georgia | 0.010463 |
| 66 | Germany | -0.192010 |
| 67 | Ghana | 0.139080 |
| 68 | Greece | 0.128260 |
| 69 | Grenada | 0.000070 |
| 70 | Guatemala | 0.333440 |
| 71 | Guinea | 0.081112 |
| 72 | Guinea-Bissau | 0.024611 |
| 73 | Guyana | 0.193320 |
| 74 | Haiti | 0.000431 |
| 75 | Honduras | 0.099734 |
| 76 | Hungary | 0.023318 |
| 77 | Iceland | 0.031517 |
| 78 | India | 1.138900 |
| 79 | Indonesia | 0.679760 |
| 80 | Iran | -0.059262 |
| 81 | Iraq | -0.023304 |
| 82 | Ireland | -0.029814 |
| 83 | Israel | -0.000832 |
| 84 | Italy | -0.023779 |
| 85 | Jamaica | 0.001328 |
| 86 | Japan | 0.253360 |
| 87 | Jordan | 0.000332 |
| 88 | Kazakhstan | -0.260470 |
| 89 | Kenya | 1.098600 |
| 90 | Kiribati | 0.000047 |
| 91 | Kuwait | -0.001907 |
| 92 | Kyrgyzstan | -0.004415 |
| 93 | Laos | -0.036392 |
| 94 | Latvia | 0.039671 |
| 95 | Lebanon | -0.001073 |
| 96 | Lesotho | 0.025499 |
| 97 | Liberia | 0.041169 |
| 98 | Libya | -0.033517 |
| 99 | Liechtenstein | 0.000720 |
| 100 | Lithuania | 0.055370 |
| 101 | Luxembourg | -0.002734 |
| 102 | Madagascar | 0.186010 |
| 103 | Malawi | 0.032910 |
| 104 | Malaysia | 0.631710 |
| 105 | Maldives | NA |
| 106 | Mali | 0.421330 |
| 107 | Malta | 0.000013 |
| 108 | Marshall Islands | NA |
| 109 | Mauritania | 0.133100 |
| 110 | Mauritius | 0.000143 |
| 111 | Mexico | -0.245030 |
| 112 | Micronesia (Federated States of) | 0.000006 |
| 113 | Moldova | -0.007346 |
| 114 | Monaco | NA |
| 115 | Mongolia | 0.169380 |
| 116 | Montenegro | 0.012447 |
| 117 | Morocco | 0.134950 |
| 118 | Mozambique | 0.356570 |
| 119 | Myanmar | -0.032568 |
| 120 | Namibia | 0.542670 |
| 121 | Nauru | NA |
| 122 | Nepal | 0.033375 |
| 123 | Netherlands | -0.001912 |
| 124 | New Zealand | 0.502360 |
| 125 | Nicaragua | 0.295260 |
| 126 | Niger | 0.154310 |
| 127 | Nigeria | 0.736110 |
| 128 | Niue | -0.000062 |
| 129 | North Korea | 0.037505 |
| 130 | North Macedonia | 0.019198 |
| 131 | Norway | 0.142280 |
| 132 | Oman | -0.003020 |
| 133 | Pakistan | 0.108950 |
| 134 | Palau | 0.000018 |
| 135 | Panama | 0.120840 |
| 136 | Papua New Guinea | 0.535760 |
| 137 | Paraguay | 0.169820 |
| 138 | Peru | 1.310300 |
| 139 | Philippines | 0.325390 |
| 140 | Poland | 0.056591 |
| 141 | Portugal | 0.031131 |
| 142 | Qatar | -0.000288 |
| 143 | Romania | 0.073427 |
| 144 | Russia | 5.932900 |
| 145 | Rwanda | 0.059404 |
| 146 | Saint Kitts and Nevis | 0.000006 |
| 147 | Saint Lucia | 0.000043 |
| 148 | Saint Vincent and the Grenadines | 0.000141 |
| 149 | Samoa | 0.000706 |
| 150 | San Marino | -0.001837 |
| 151 | Sao Tome and Principe | 0.000022 |
| 152 | Saudi Arabia | -0.052402 |
| 153 | Senegal | 0.118090 |
| 154 | Serbia | 0.060398 |
| 155 | Seychelles | 0.000006 |
| 156 | Sierra Leone | -0.068552 |
| 157 | Singapore | 0.001331 |
| 158 | Slovakia | -0.006569 |
| 159 | Slovenia | -0.008071 |
| 160 | Solomon Islands | 0.004413 |
| 161 | Somalia | 0.191110 |
| 162 | South Africa | 0.590310 |
| 163 | South Korea | 0.053471 |
| 164 | South Sudan | 0.829530 |
| 165 | Spain | 0.180920 |
| 166 | Sri Lanka | 0.038524 |
| 167 | State of Palestine | 0.000185 |
| 168 | Sudan | 0.718300 |
| 169 | Suriname | 0.152800 |
| 170 | Sweden | 0.221620 |
| 171 | Switzerland | 0.028171 |
| 172 | Syria | -0.000737 |
| 173 | Tajikistan | -0.002093 |
| 174 | Tanzania | 0.478680 |
| 175 | Thailand | 0.655690 |
| 176 | Timor-Leste | -0.007165 |
| 177 | Togo | 0.023000 |
| 178 | Tonga | 0.000037 |
| 179 | Trinidad and Tobago | 0.007056 |
| 180 | Tunisia | 0.005328 |
| 181 | Türkiye | 0.297720 |
| 182 | Turkmenistan | -0.009710 |
| 183 | Tuvalu | NA |
| 184 | Uganda | 0.333990 |
| 185 | Ukraine | -0.196520 |
| 186 | United Arab Emirates | 0.000323 |
| 187 | United Kingdom | -0.025449 |
| 188 | Uruguay | -0.033184 |
| 189 | USA | 0.933230 |
| 190 | Uzbekistan | -0.031019 |
| 191 | Vanuatu | 0.001293 |
| 192 | Vatican City | NA |
| 193 | Venezuela | 0.119170 |
| 194 | Viet Nam | -0.804150 |
| 195 | Yemen | 0.039506 |
| 196 | Zambia | 1.019200 |
| 197 | Zimbabwe | 0.551550 |

^a^The trend of land carbon sink in China is calculated using the net biome production (NBP) of the S3 experiment from this study. Datasets for other country/region are based on the NBP of the S3 experiment in the Global Carbon Budget 2023. NA indicates that the data is not available. The country/region names follow the Global Carbon Budget 2023 [49].

# Table S4. Comparison of previous terrestrial carbon sink estimates in China with this study

| **References** | **Period** | **Method** | **Previous study**  **(PgC yr^-1^)** | **This study**  **(PgC yr^-1^)** |
| --- | --- | --- | --- | --- |
| Piao et al. 2009[63] | 1980−1999 | Inventory method | 0.187 | 0.055 |
| Fang et al. 2018[18] | 2001−2010 | Inventory method | 0.200 | 0.257 |
| Jiang et al. 2016[64] | 2000−2009 | Inventory method | 0.329 | 0.247 |
| Piao et al. 2009[63] | 1980−2002 | Ecosystem process model | 0.174 | 0.073 |
| He et al. 2019[65] | 1982−2010 | Ecosystem process model | 0.116 | 0.132 |
| Friedlingstein et al. 2020[66] | 2010−2017 | Ecosystem process model | 0.264 | 0.300 |
| Tian et al. 2011[67] | 1981−2000 | Ecosystem process model | 0.120 | 0.058 |
| Tian et al. 2011[67] | 1996−2005 | Ecosystem process model | 0.260 | 0.205 |
| Tian et al. 2011[67] | 2000−2005 | Ecosystem process model | 0.290 | 0.228 |
| Yu et al. 2022[58] | 1980−2010 | Ecosystem process model | 0.210 | 0.136 |
| Yu et al. 2022[58] | 2000−2010 | Ecosystem process model | 0.300 | 0.258 |
| Yu et al. 2022[58] | 2010−2019 | Ecosystem process model | 0.280 | 0.319 |
| Yu et al. 2022[58] | 1980−2019 | Ecosystem process model | 0.230 | 0.180 |
| Xia et al. 2024[9] | 2010−2021 | Ecosystem process model | 0.450 | 0.313 |
| Chen et al. 2019[19] | 2010−2015 | Ecosystem process model | 0.437 | 0.306 |
| Piao et al. 2009[63] | 1996−2005 | Atmospheric inversion method | 0.258 | 0.200 |
| Jiang et al. 2016[64] | 2006−2009 | Atmospheric inversion method | 0.348 | 0.276 |
| Wang et al. 2022[68] | 2010−2016 | Atmospheric inversion method | 0.252 | 0.301 |
| Peters et al. 2007[69] | 2000−2005 | Atmospheric inversion method | 0.260 | 0.228 |
| Zhang et al. 2014[70] | 2001−2010 | Atmospheric inversion method | 0.335 | 0.257 |
| Thompson et al. 2016[71] | 2001−2005 | Atmospheric inversion method | 0.330 | 0.255 |
| Friedlingstein et al. 2020[66] | 2010−2016 | Atmospheric inversion method | 0.309 | 0.301 |
| Friedlingstein et al. 2020[66] | 2010−2016 | Atmospheric inversion method | 0.342 | 0.301 |
| Friedlingstein et al. 2020[66] | 2010−2016 | Atmospheric inversion method | 0.348 | 0.301 |
| Chen et al. 2021[72] | 2010−2013 | Atmospheric inversion method | 0.445 | 0.303 |

# References

1. Harris I, Jones PD, Osborn TJ *et al.* Updated high-resolution grids of monthly climatic observations – the CRU TS3.10 Dataset. *Int J Climatol* 2014; **34**: 623-42.

2. Harris I, Osborn TJ, Jones P *et al.* Version 4 of the CRU TS monthly high-resolution gridded multivariate climate dataset. *Sci Data* 2020; **7**: 109.

3. Harris IC. CRU JRA v2.4: A forcings dataset of gridded land surface blend of Climatic Research Unit (CRU) and Japanese reanalysis (JRA) data; Jan.1901 - Dec.2022. *NERC EDS Centre for Environmental Data Analysis* 2023.

4. Kobayashi S, Ota Y, Harada Y *et al.* The JRA-55 Reanalysis: General Specifications and Basic Characteristics. *J Meteorolog Soc Jpn Ser II* 2015; **93**: 5-48.

5. Muñoz-Sabater J, Dutra E, Agustí-Panareda A *et al.* ERA5-Land: a state-of-the-art global reanalysis dataset for land applications. *Earth Syst Sci Data* 2021; **13**: 4349-83.

6. Yuan *et al*. China's greenhouse gas budget during 2000-2023. *Under review in this issue*.

7. Xia X, Xia J, Chen X *et al.* Reconstructing Long-Term Forest Cover in China by Fusing National Forest Inventory and 20 Land Use and Land Cover Data Sets. *J Geophys Res: Biogeosci* 2023; **128**: e2022JG007101.

8. Yang J, Huang X. The 30m annual land cover dataset and its dynamics in China from 1990 to 2019. *Earth Syst Sci Data* 2021; **13**: 3907-25.

9. Xia X, Ren P, Wang X *et al.* The carbon budget of China: 1980–2021. *Sci Bull* 2024; **69**: 114-24.

10. Hurtt GC, Chini L, Sahajpal R *et al.* Harmonization of global land use change and management for the period 850–2100 (LUH2) for CMIP6. *Geosci Model Dev* 2020; **13**: 5425-64.

11. National Development and Reform Commission. *The People’s Republic of China Fourth National Communication on Climate Change (in Chinese)*. 2023.

12. Ministry of Ecology and Environment of the People’s Republic of China. *The People’s Republic of China Third Biennial Update Report on Climate Change (in Chinese)*. 2023.

13. Ministry of Ecology and Environment of the People’s Republic of China. *The People’s Republic of China Third National Communication on Climate Change (in Chinese)*. 2018.

14. Ministry of Ecology and Environment of the People’s Republic of China. *The People’s Republic of China Second Biennial Update Report on Climate Change (in Chinese)*. 2019.

15. National Development and Reform Commission. *The People’s Republic of China Initial National Communication on Climate Change (in Chinese)*. 2004.

16. National Development and Reform Commission. *The People’s Republic of China First Biennial Update Report on Climate Change (in Chinese)*. 2016.

17. National Development and Reform Commission. *The People’s Republic of China Second National Communication on Climate Change (in Chinese)*. 2013.

18. Fang J, Yu G, Liu L *et al.* Climate change, human impacts, and carbon sequestration in China. *Proc Natl Acad Sci USA* 2018; **115**: 4015-20.

19. Chen JM, Ju W, Ciais P *et al.* Vegetation structural change since 1981 significantly enhanced the terrestrial carbon sink. *Nat Commun* 2019; **10**: 4259.

20. Farquhar GD, von Caemmerer S,Berry JA. A biochemical model of photosynthetic CO2 assimilation in leaves of C3 species. *Planta* 1980; **149**: 78-90.

21. Chen JM, Liu J, Cihlar J *et al.* Daily canopy photosynthesis model through temporal and spatial scaling for remote sensing applications. *Ecol Modell* 1999; **124**: 99-119.

22. Foley JA, Prentice IC, Ramankutty N *et al.* An integrated biosphere model of land surface processes, terrestrial carbon balance, and vegetation dynamics. *Global Biogeochem Cy* 1996; **10**: 603-28.

23. Xia J, Chen Y, Liang S *et al.* Global simulations of carbon allocation coefficients for deciduous vegetation types. *Tellus B: Chem Phys Meteorol* 2015; **67**: 28016.

24. Zhang H, Liu S, Regnier P *et al.* New insights on plant phenological response to temperature revealed from long-term widespread observations in China. *Global Change Biol* 2018; **24**: 2066-78.

25. Lu H, Yuan W,Chen X. A Processes-Based Dynamic Root Growth Model Integrated Into the Ecosystem Model. *J Adv Model Earth Syst* 2019; **11**: 4614-28.

26. Song C, Luan J, Xu X *et al.* A Microbial Functional Group-Based CH_4_ Model Integrated Into a Terrestrial Ecosystem Model: Model Structure, Site-Level Evaluation, and Sensitivity Analysis. *J Adv Model Earth Syst* 2020; **12**: e2019MS001867.

27. Ma M, Song C, Fang H *et al.* Development of a Process-Based N_2_O Emission Model for Natural Forest and Grassland Ecosystems. *J Adv Model Earth Syst* 2022; **14**: e2021MS002460.

28. Liu D, Chen Y, Cai W *et al.* The contribution of China’s Grain to Green Program to carbon sequestration. *Landscape Ecol* 2014; **29**: 1675-88.

29. Yuan W, Liu D, Dong W *et al.* Multiyear precipitation reduction strongly decreases carbon uptake over northern China. *J Geophys Res: Biogeosci* 2014; **119**: 881-96.

30. Yue X,Unger N. The Yale Interactive terrestrial Biosphere model version 1.0: description, evaluation and implementation into NASA GISS ModelE2. *Geosci Model Dev* 2015; **8**: 2399-417.

31. Yue X, Zhou H, Tian C *et al.* Development and evaluation of the interactive Model for Air Pollution and Land Ecosystems (iMAPLE) version 1.0. *Geosci Model Dev* 2024; **17**: 4621-42.

32. Spitters CJT, Toussaint HAJM,Goudriaan J. Separating the diffuse and direct component of global radiation and its implications for modeling canopy photosynthesis Part I. Components of incoming radiation. *Agric For Meteorol* 1986; **38**: 217-29.

33. Niu G-Y, Yang Z-L, Mitchell KE *et al.* The community Noah land surface model with multiparameterization options (Noah-MP): 1. Model description and evaluation with local-scale measurements. *J Geophys Res: Atmos* 2011; **116**.

34. Ball JT, Woodrow IE,Berry JA. A Model Predicting Stomatal Conductance and its Contribution to the Control of Photosynthesis under Different Environmental Conditions. In: Biggins J (ed.) *Progress in Photosynthesis Research: Volume 4 Proceedings of the VIIth International Congress on Photosynthesis Providence, Rhode Island, USA, August 10–15, 1986*. Dordrecht: Springer Netherlands; 1987. 221-4.

35. Smith B, Wårlind D, Arneth A *et al.* Implications of incorporating N cycling and N limitations on primary production in an individual-based dynamic vegetation model. *Biogeosciences* 2014; **11**: 2027-54.

36. Olin S, Schurgers G, Lindeskog M *et al.* Modelling the response of yields and tissue C : N to changes in atmospheric CO_2_ and N management in the main wheat regions of western Europe. *Biogeosciences* 2015; **12**: 2489-515.

37. Lindeskog M, Smith B, Lagergren F *et al.* Accounting for forest management in the estimation of forest carbon balance using the dynamic vegetation model LPJ-GUESS (v4.0, r9710): implementation and evaluation of simulations for Europe. *Geosci Model Dev* 2021; **14**: 6071-112.

38. Sitch S, O’Sullivan M, Robertson E *et al.* Trends and Drivers of Terrestrial Sources and Sinks of Carbon Dioxide: An Overview of the TRENDY Project. *Global Biogeochem Cy* 2024; **38**: e2024GB008102.

39. Krinner G, Viovy N, de Noblet-Ducoudré N *et al.* A dynamic global vegetation model for studies of the coupled atmosphere-biosphere system. *Global Biogeochem Cy* 2005; **19**.

40. Yin Z, Wang XH, Ottlé C *et al.* Improvement of the Irrigation Scheme in the ORCHIDEE Land Surface Model and Impacts of Irrigation on Regional Water Budgets Over China. *J Adv Model Earth Syst* 2020; **12**: e2019MS001770.

41. Guimberteau M, Zhu D, Maignan F *et al.* ORCHIDEE-MICT (v8.4.1), a land surface model for the high latitudes: model description and validation. *Geosci Model Dev* 2018; **11**: 121-63.

42. Liu J, Lu X, Zhu Q *et al.* Terrestrial Ecosystem Modeling with IBIS: Progress and Future Vision. *J Resour Ecol* 2022; **13**: 2-16, 5.

43. Zhang K, Peng C, Wang M *et al.* Process-based TRIPLEX-GHG model for simulating N_2_O emissions from global forests and grasslands: Model development and evaluation. *J Adv Model Earth Syst* 2017; **9**: 2079-102.

44. Zhu Q, Chen H, Peng C *et al.* An early warning signal for grassland degradation on the Qinghai-Tibetan Plateau. *Nat Commun* 2023; **14**: 6406.

45. Zhu Q, Liu J, Peng C *et al.* Modelling methane emissions from natural wetlands by development and application of the TRIPLEX-GHG model. *Geosci Model Dev* 2014; **7**: 981-99.

46. Kucharik CJ, Foley JA, Delire C *et al.* Testing the performance of a dynamic global ecosystem model: Water balance, carbon balance, and vegetation structure. *Global Biogeochem Cy* 2000; **14**: 795-825.

47. Liu J, Price DT,Chen JM. Nitrogen controls on ecosystem carbon sequestration: a model implementation and application to Saskatchewan, Canada. *Ecol Modell* 2005; **186**: 178-95.

48. Liu J, Sleeter BM, Zhu Z *et al.* Critical land change information enhances the understanding of carbon balance in the United States. *Global Change Biol* 2020; **26**: 3920-9.

49. Friedlingstein P, O'Sullivan M, Jones MW *et al.* Global Carbon Budget 2023. *Earth Syst Sci Data* 2023; **15**: 5301-69.

50. Zhang H, Lauerwald R, Ciais P *et al.* Global changes alter the amount and composition of land carbon deliveries to European rivers and seas. *Commun Earth Environ* 2022; **3**: 245.

51. Zhang H, Lauerwald R, Regnier P *et al.* Estimating the lateral transfer of organic carbon through the European river network using a land surface model. *Earth Syst Dynam* 2022; **13**: 1119-44.

52. Lin Z, Huang L, Tian H *et al.* China Wildfire Emission (ChinaWED v1) for the period 2012–2022. *Geosci Model Dev Discuss* 2024; **in review**: 1-21.

53. Schroeder W, Oliva P, Giglio L *et al.* The New VIIRS 375m active fire detection data product: Algorithm description and initial assessment. *Remote Sens Environ* 2014; **143**: 85-96.

54. Di Giuseppe F, Benedetti A, Coughlan R *et al.* A Global Bottom-Up Approach to Estimate Fuel Consumed by Fires Using Above Ground Biomass Observations. *Geophys Res Lett* 2021; **48**: e2021GL095452.

55. Qin Z, Zhu Y, Canadell JG *et al.* Global spatially explicit carbon emissions from land-use change over the past six decades (1961–2020). *One Earth* 2024; **7**: 835-47.

56. Zhu Y, Xia X, Canadell JG *et al.* China's carbon sinks from land-use change underestimated. *Nat Clim Change* 2025; accepted.

57. O’Sullivan M, Zhang Y, Bellouin N *et al.* Aerosol–light interactions reduce the carbon budget imbalance. *Environ Res Lett* 2021; **16**: 124072.

58. Yu Z, Ciais P, Piao S *et al.* Forest expansion dominates China’s land carbon sink since 1980. *Nat Commun* 2022; **13**: 5374.

59. Hansis E, Davis SJ,Pongratz J. Relevance of methodological choices for accounting of land use change carbon fluxes. *Global Biogeochem Cy* 2015; **29**: 1230-46.

60. Gasser T, Crepin L, Quilcaille Y *et al.* Historical CO_2_ emissions from land use and land cover change and their uncertainty. *Biogeosciences* 2020; **17**: 4075-101.

61. Houghton RA,Castanho A. Annual emissions of carbon from land use, land-use change, and forestry from 1850 to 2020. *Earth Syst Sci Data* 2023; **15**: 2025-54.

62. Bastos A, Hartung K, Nützel TB *et al.* Comparison of uncertainties in land-use change fluxes from bookkeeping model parameterisation. *Earth Syst Dynam* 2021; **12**: 745-62.

63. Piao S, Fang J, Ciais P *et al.* The carbon balance of terrestrial ecosystems in China. *Nature* 2009; **458**: 1009-13.

64. Jiang F, Chen JM, Zhou L *et al.* A comprehensive estimate of recent carbon sinks in China using both top-down and bottom-up approaches. *Sci Rep* 2016; **6**: 22130.

65. He H, Wang S, Zhang L *et al.* Altered trends in carbon uptake in China's terrestrial ecosystems under the enhanced summer monsoon and warming hiatus. *Natl Sci Rev* 2019; **6**: 505-14.

66. Friedlingstein P, O'Sullivan M, Jones MW *et al.* Global Carbon Budget 2020. *Earth Syst Sci Data* 2020; **12**: 3269-340.

67. Tian H, Melillo J, Lu C *et al.* China's terrestrial carbon balance: Contributions from multiple global change factors. *Global Biogeochem Cy* 2011; **25**.

68. Wang Y, Wang X, Wang K *et al.* The size of the land carbon sink in China. *Nature* 2022; **603**: E7-E9.

69. Peters W, Jacobson AR, Sweeney C *et al.* An atmospheric perspective on North American carbon dioxide exchange: CarbonTracker. *Proc Natl Acad Sci USA* 2007; **104**: 18925-30.

70. Zhang HF, Chen BZ, van der Laan-Luijkx IT *et al.* Net terrestrial CO_2_ exchange over China during 2001–2010 estimated with an ensemble data assimilation system for atmospheric CO_2_. *J Geophys Res: Atmos* 2014; **119**: 3500-15.

71. Thompson RL, Patra PK, Chevallier F *et al.* Top–down assessment of the Asian carbon budget since the mid 1990s. *Nat Commun* 2016; **7**: 10724.

72. Chen B, Zhang H, Wang T *et al.* An atmospheric perspective on the carbon budgets of terrestrial ecosystems in China: progress and challenges. *Sci Bull* 2021; **66**: 1713-8.
